# Temperate species underfill their tropical thermal potentials on land

**DOI:** 10.1038/s41559-023-02239-x

**Published:** 2023-11-06

**Authors:** Nikki A. Moore, Ignacio Morales-Castilla, Anna L. Hargreaves, Miguel Ángel Olalla-Tárraga, Fabricio Villalobos, Piero Calosi, Susana Clusella-Trullas, Juan G. Rubalcaba, Adam C. Algar, Brezo Martínez, Laura Rodríguez, Sarah Gravel, Joanne M. Bennett, Greta C. Vega, Carsten Rahbek, Miguel B. Araújo, Joey R. Bernhardt, Jennifer M. Sunday

**Affiliations:** 1 Department of Biology, McGill University, Montreal, Quebec Canada; 2 Department of Life Sciences, Global Change Ecology and Evolution Group, Universidad de Alcalá; Alcalá de Henares, Madrid, Spain; 3 Departamento de Biología y Geología, Física y Química Inorgánica, Universidad Rey Juan Carlos, Móstoles, Spain; 4 Red de Biología Evolutiva, Instituto de Ecología A. C, Xalapa, Mexico; 5 Marine Ecological and Evolutionary Physiology Laboratory, Département de Biologie, Chimie et Géographie, Université du Québec à Rimouski, Rimouski, Quebec Canada; 6 Department of Botany and Zoology and School for Climate Studies, Stellenbosch University, Stellenbosch, South Africa; 7 Department of Biology, Lakehead University, Thunder Bay, Ontario Canada; 8 Department of Biology (Grupo en Biodiversidad y Conservación, IU-ECOAQUA), Marine Sciences Faculty, University of Las Palmas de Gran Canaria; Las Palmas de G.C., Canary Islands, Spain; 9 Department of Biological Sciences, Simon Fraser University, Burnaby, British Columbia Canada; 10 Fenner School of Environment & Society, College of Science, The Australian National University, Canberra, Australian Capital Territory Australia; 11 Center for Global Mountain Biodiversity, GLOBE Institute, University of Copenhagen, Copenhagen, Denmark; 12 Center for Macroecology, Evolution and Climate, GLOBE Institute, University of Copenhagen, Copenhagen, Denmark; 13 Institute of Ecology, Peking University, Beijing, China; 14 Danish Institute for Advanced Study, University of Southern Denmark, Odense M, Denmark; 15 Department of Biogeography and Global Change, National Museum of Natural Sciences, CSIC, Madrid, Spain; 16 ‘Rui Nabeiro’ Biodiversity Chair, MED Institute, University of Évora, Évora, Portugal; 17 Department of Integrative Biology, University of Guelph, Guelph, Ontario Canada

**Keywords:** Biogeography, Macroecology, Evolutionary theory, Climate-change ecology, Evolutionary ecology

## Abstract

Understanding how temperature determines the distribution of life is necessary to assess species’ sensitivities to contemporary climate change. Here, we test the importance of temperature in limiting the geographic ranges of ectotherms by comparing the temperatures and areas that species occupy to the temperatures and areas species could potentially occupy on the basis of their physiological thermal tolerances. We find that marine species across all latitudes and terrestrial species from the tropics occupy temperatures that closely match their thermal tolerances. However, terrestrial species from temperate and polar latitudes are absent from warm, thermally tolerable areas that they could potentially occupy beyond their equatorward range limits, indicating that extreme temperature is often not the factor limiting their distributions at lower latitudes. This matches predictions from the hypothesis that adaptation to cold environments that facilitates survival in temperate and polar regions is associated with a performance trade-off that reduces species’ abilities to contend in the tropics, possibly due to biotic exclusion. Our findings predict more direct responses to climate warming of marine ranges and cool range edges of terrestrial species.

## Abstract (web summary)

The authors test whether a wide array of marine and terrestrial animal species occupy the full extent of their potential geographic range based on thermal tolerances. They find that many species are underfilling the warm part of their potential range, suggesting that biotic interactions can limit occupancy in climatically suitable areas adjacent to their ranges.

## Main

Climate warming is already altering the distributions of species worldwide^1^. Yet sensitivity of biogeographic distributions to climate change varies considerably among species^2,3^, calling into question how consistently temperature limits geographic ranges. If a species occupies all accessible habitat where temperatures suit its tolerances (that is, the species fills its potential thermal niche), then the species’ range limits are expected to be sensitive to temperature change^4^. Yet, in reality, species are often unable to fill their potential thermal niche when ranges are limited by other factors^5–7^, such as dispersal^8^, species interactions^9^, resource availability^9^ and non-thermal abiotic factors like moisture (on land)^10^ or oxygen (in water)^11^. Understanding where and when temperature directly constrains species ranges can help clarify the mechanisms responsible for historical range shifts and improve projections of species’ sensitivities to contemporary climate warming.

A relevant long-standing hypothesis posits that temperature and other abiotic factors are more limiting at species’ poleward range edges compared to their equatorward edges^12,13^. This is supported by a recent synthesis of empirical studies of the ecological factors limiting species ranges, which shows that biotic interactions influence species’ low-latitude and low-elevation range edges more often than their high-latitude and high-elevation edges^9^. One possible cause of this pattern, supported by recent empirical evidence^14–18^, is that antagonistic species interactions become more intense toward the tropics^9,12,13^ owing to the increased biodiversity, density or activity levels in the more productive, warmer and more seasonally stable tropics (reviewed by ref. ^19^).

However, the mechanisms by which antagonistic biotic interactions become more important toward lower latitudes remain unclear and two particularly relevant hypotheses offer testable predictions. The first posits that the role of abiotic factors in limiting species ranges gradually decreases towards lower latitudes because of the increasing intensity and importance of antagonistic biotic interactions, such as interspecific competition, parasitism or predation^14–17^. Under this ‘reduced-abiotic-limitation-in-the-tropics hypothesis’, all range limits toward lower latitudes (both poleward and equatorward range limits that occur at low latitudes) are expected to be less abiotically limited than those at higher latitudes.

The second hypothesis posits that evolution of greater cold tolerance needed to persist outside the tropics comes at the cost of withstanding natural enemies at warmer latitudes, resulting in biotic exclusion. High-latitude species have adapted to endure environments with both colder temperature extremes and greater seasonal temperature fluctuations^20^, leading them to have wider temperature tolerance breadths than tropical species^21^. Yet greater tolerance to colder, more thermally variable environments is thought to come at the cost of lower performance in warmer temperatures due to a specialist–generalist trade-off^22–25^ (‘jack-of-all-trades is master of none’, principle of allocation^26,27^). This trade-off might cause higher-latitude species to have lower resistance to antagonistic biotic interactions compared to lower latitude species, which could lead to biotic exclusion of higher-latitude species at their equatorward range edges where they are outperformed by their tropical counterparts. We call this the ‘temperate-trade-off hypothesis’.

**Fig. 1:**
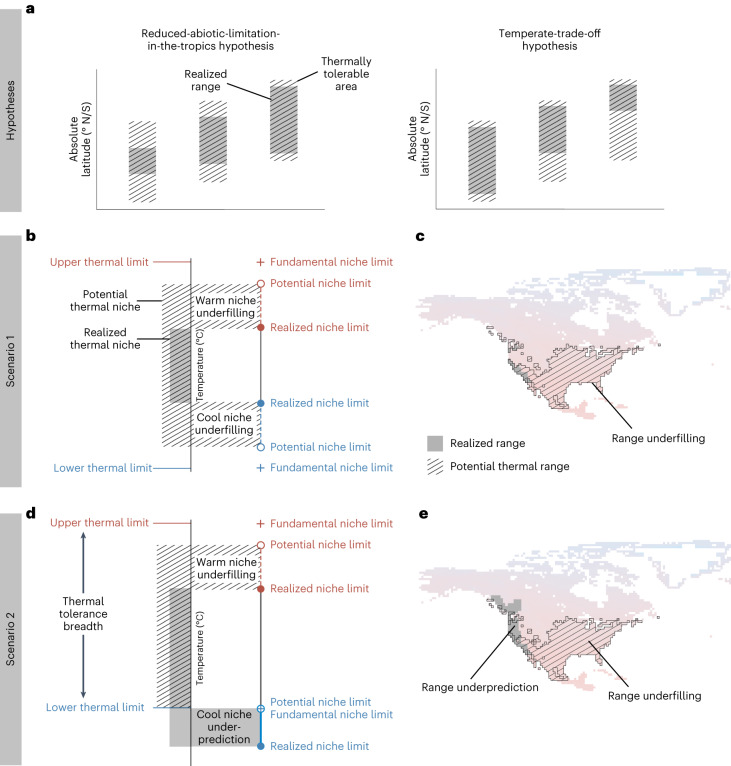
Predictions and definitions of thermal niche filling projected in thermal and geographic space. **a**, The reduced-abiotic-limitations-in-the-tropics hypothesis (left) predicts that stronger antagonistic species interactions in the tropics will exclude lower latitude species from occupying more thermally tolerable habitat at either range edge compared to higher-latitude species, while the temperate-trade-off hypothesis (right) predicts that a trade-off between cold adaptation and performance will cause higher-latitude species to be excluded from thermally tolerable habitat towards the equator. **b**–**e**, Two explanatory scenarios (1, **b** and **c**; 2, **d** and **e**) illustrate potential and realized overlap in thermal space (**b** and **d**) and geographic space (**c** and **e**). **b**,**d**, The fundamental thermal niche is defined by the physiologically determined maximum (red) and minimum (blue) thermal tolerance limits, the difference between which defines a species’ thermal tolerance breadth. A species’ potential thermal niche is the extreme body temperatures within its fundamental thermal niche that it can experience (given constrained thermoregulatory behaviour) across the encounterable habitat (here defined as the landscape or seascape contiguous with the species’ realized range). A species’ realized thermal niche is the extreme body temperatures it can experience throughout its realized range. Potential thermal niche limits differ from fundamental thermal limits when temperatures within the fundamental niche are not found in the current climate across the accessible habitat. **c**,**e**, A species’ realized range encloses its observed extent of geographic occurrence, while its potential thermal range encloses the areas of available habitat where extreme body temperatures remain within the species’ fundamental thermal niche limits. A species might not occur in all available niche space (niche underfilling, **a**; range underfilling, **b**) or might appear to occur beyond the available niche space if its thermal tolerance limits underpredict its geographic distribution (niche underprediction, **c**; range underprediction, **d**).

While not mutually exclusive, these two hypotheses make contrasting and testable predictions about how species interactions might alter the relative importance of temperature in limiting ranges. The reduced-abiotic-limitation-in-the-tropics hypothesis predicts that all range edges are increasingly biotically constrained toward the equator, such that species are more excluded from environments with tolerable temperatures toward lower latitudes. The temperate-trade-off hypothesis predicts greater biotic exclusion of higher-latitude species at their equatorward range edges specifically, where they are outperformed by lower latitude species. Thus, while both hypotheses assume that biotic exclusion increases towards lower latitudes, as supported by increasing strength of biotic interactions towards the equator^9,12,13^, they differ in whether the predicted asymmetry in exclusion occurs across absolute latitudes or within each species range (Fig. 1a).

Species range limits might be set by factors other than biotic interactions, which could add variation in the predicted latitudinal patterns of biotic exclusion. For example, species with poor dispersal ability might be more out-of-equilibrium with temperatures in the current climate^4^ (for example, climate disequilibrium in tree species rebounding from the last glacial maximum^28–30^), so their ranges might be limited less directly by temperature. In addition, ecologically specialized species could be more limited by other constraints^31^ (for example, availability of a specific food resource or specific type of habitats), resulting in their ranges being limited less directly by temperature. Finally, species differ in their ability to avoid extreme temperatures. Those with greater capacity to physiologically adjust to temperature variation or behaviourally thermoregulate are expected to have ranges that are less directly limited by temperature extremes. We codified these variables as traits (Extended Data Table 1) and tested their effects alongside our main hypotheses.

We tested our main hypotheses in a spatially explicit global analysis of the potential and realized thermal niches of ectotherms. We used critical and lethal thermal tolerance limits from experimental assays to define the fundamental thermal niche (Fig. 1b–e). In current climates, some temperatures within a species’ fundamental thermal niche might not occur anywhere on Earth^32–35^ or might only occur far outside the species’ current distribution. To describe the tolerable thermal niche space available to a species given climatic and geographic constraints, we delineated the potential thermal niche as the ‘encounterable’ warm and cool extreme body temperatures within the fundamental thermal niche (Methods). We assessed how well species fill their potential thermal niches by comparing each species’ potential thermal niche to its realized thermal niche, defined by the warm and cool extreme body temperatures across its current estimated geographic distribution (that is, its realized range; see Supplementary Methods Section 1 for technical definitions of fundamental, potential and realized thermal niche in the sense of ref. ^36^). We measured thermal niche filling as the difference between the potential and realized thermal niche extremes. Negative values denote niche underfilling (cases in which species do not occupy all extreme temperatures available to them, in the sense of ref. ^37^; Fig. 1b,d) and positive values denote niche underprediction (cases in which species’ thermal tolerance limits are narrower than the extreme body temperatures they are expected to encounter across their current geographic distributions; Fig. 1b,d).

Whereas thermal niche filling describes offsets between occupied and tolerable temperatures, how these offsets play out in geographic space depends on the spatial distribution of temperature^38^ (Fig. 1c,e). To test between our two main hypotheses, we also assessed how patterns of potential thermal niche filling differ when analysed in geographic space (Fig. 1c,e). We measured range filling as the proportion of a species’ potential thermal range that it occupies and assessed whether range underfilling was biased towards species’ equatorward or poleward range edges.

We used latitudinal patterns in niche filling and range filling to test the alternative expectations from the temperate-trade-off hypothesis and the reduced-abiotic-limitation-in-the-tropics hypothesis. We tested our hypotheses within terrestrial, intertidal and subtidal marine realms under the expectation that thermal niche filling differs between marine and terrestrial environments^3,37,39^. We also asked whether species with lower dispersal potential, with lower capacity to thermoregulate or that are more ecologically specialized have greater niche or range underfilling. Additionally, we assessed the sensitivity of latitudinal relationships to variation in thermal tolerance and encountered temperatures owing to species’ capacity to behaviourally thermoregulate and to adjust their thermal limits through acclimatization (Methods).

## Results and discussion

**Fig. 2:**
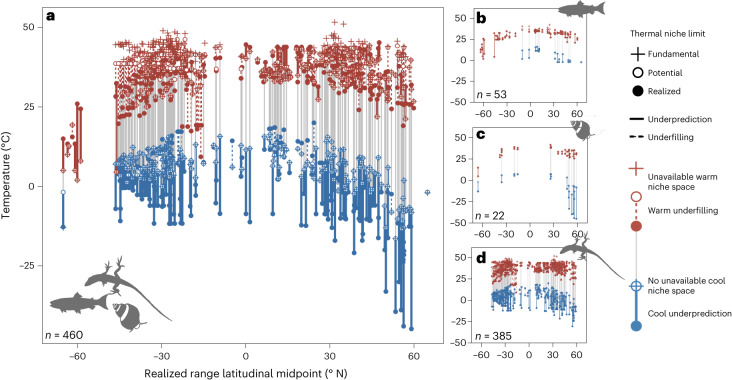
Species underfill their warm thermal niche and cold thermal tolerance limits underpredict their cool thermal niche. **a**–**d**, Species’ fundamental (crosses), realized (solid circles) and potential (open circles) thermal niche limits versus the latitudinal midpoint of their realized range for all realms (**a**) and separately for subtidal marine (**b**), intertidal marine (**c**) and terrestrial (**d**) species. Warm and cool niche limits are shown in red and blue, respectively. Dashed lines connecting the potential and realized niche limits indicate breadth of warm or cool niche underfilling, while thick connecting lines indicate breadth of warm or cool niche underprediction.

**Fig. 3:**
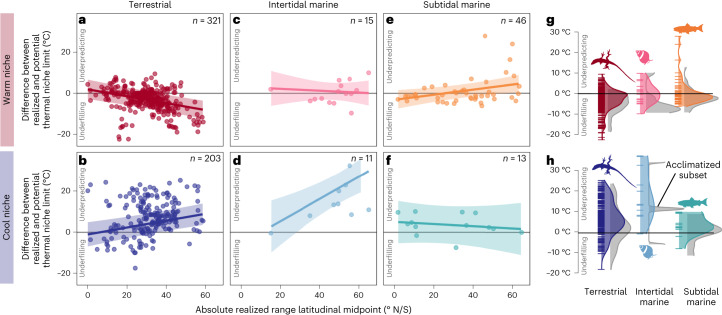
Warm and cool niche filling vary with the absolute latitude of a species’ range, realm and acclimatization. **a**–**f**, Warm (warm shades, **a**,**c**,**e**) and cool (cool shades, **b**,**d**,**f**) filling of the potential thermal niche versus the absolute latitudinal midpoint of a species’ realized range, showing model fitted relationships (lines) and associated confidence intervals (shaded areas) from separate models of warm and cool niche filling as a function of variables in Extended Data Table 1. Each point represents the shortfall (negative, underfilling) or excess (positive, underprediction) of temperatures occupied beyond the potential niche limit at either the warm or cool edge of a species’ potential thermal niche (difference between potential and realized niche limit). Warm niche underfilling and cool niche underprediction increase with latitude in terrestrial species (**a**,**b**), while only cool niche underprediction increases with latitude in intertidal marine species (**c**). In subtidal species, neither warm nor cool niche filling changes with latitude (**e**,**f**). Predictions are shown for a species with the median body and range size within each realm and with the mode thermal limit metric type and dispersal distance. (**g**,**h**). Distribution of warm (**g**) and cool (**h**) thermal niche niche filling measurements (i.e., the difference between realized and potential thermal niche limits in °C) across ecological realms with (grey) and without (coloured) simulating acclimatization to the local thermal environment (sample sizes for acclimatized distributions from left to right, top to bottom are *n* = 163, 8, 41, 117, 5 and 12; see Extended Data Fig. 4 for distributions of comparable data subsets).

**Extended Data Fig. 1:**
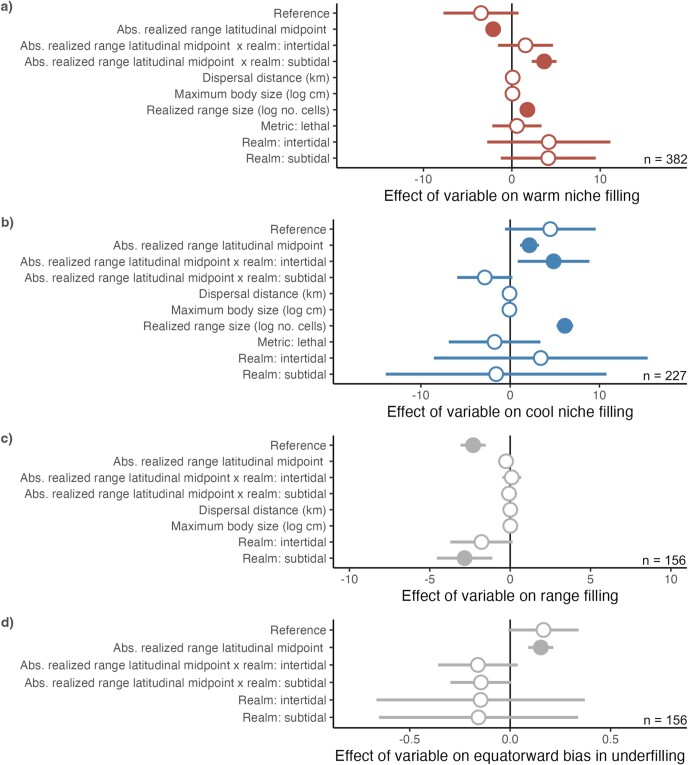
Average parameter estimates of models in confidence set for linear mixed effects models of warm and cool niche filling, potential range filling and equatorward bias in underfilling. **a–d**. Dot-and-whisker plot of average parameters and associated 95% confidence intervals of models in the 95% confidence set of linear mixed-effect models for response variables (**a**) warm niche filling (n = 382 species), (**b**) cool niche filling (n = 227 species), (**c)** range filling (n = 156 species), (**d**) equatorward bias in underfilling (n = 156 species). Reference levels for contrast coefficients of fixed effects were terrestrial for realm and critical for thermal limit metric. Solid dots denote parameter estimates whose 95% confidence intervals do not overlap zero.

Patterns of thermal niche filling in the terrestrial realm were consistent with predictions from the temperate-trade-off hypothesis. According to their thermal tolerance limits, most terrestrial species—reptiles, amphibians, insects and arachnids—could live in places with warmer extreme temperatures than those they currently experience. Hence, they underfill the warm ends of their potential thermal niche (dashed red lines in Figs. 2 and 3a). Results did not match predictions from the reduced-abiotic-limitation-in-the-tropics hypothesis, which predicts greater warm niche underfilling in species living at lower absolute latitudes. Warm niche underfilling was instead greatest in terrestrial species living farthest from the equator and increased with latitude (Figs. 2 and 3, Extended Data Fig. 1 and Extended Data Table 2). Patterns were different in the ocean; intertidal and subtidal marine species—fish and marine invertebrates—underfilled their warm thermal niche less than terrestrial species (closer to zero; that is, perfect filling) and this amount of underfilling did not change substantially with latitude (Figs. 2 and 3c,e, Extended Data Fig. 1 and Extended Data Table 2), although results are more tentative given small sample sizes from marine realms.

**Fig. 4:**
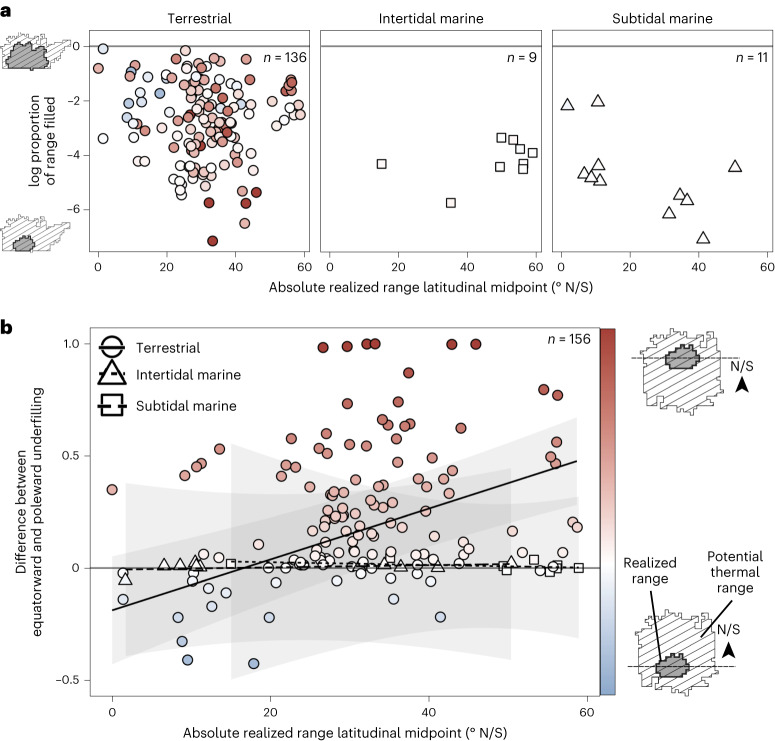
Range underfilling is biased towards the equatorward range edge of terrestrial species. **a**, Across species within all realms (shapes), the proportion of geographic area that a species occupies within its potential thermal range does not depend on the absolute latitudinal midpoint of its range. **b**, Within species, the equatorward bias of range underfilling (the difference between the proportion of a species’ equatorward and poleward potential range that is underfilled) increases with the absolute latitudinal midpoint of the species’ range in terrestrial species. Positive values (red) indicate equatorward bias, meaning underfilling is greater in the equatorward range half of the species’ range. Negative values (blue) indicate underfilling is greater in the species’ poleward range half. Intertidal and subtidal marine species across all latitudes show very little equatorward bias in underfilling (white points). Lines show best-fit relationships and shaded areas indicate 95% confidence intervals from the model of equatorward bias in range underfilling as a function of variables in Extended Data Table 1. Predictions are shown for a species with the median body size and the mode thermal limit metric type and dispersal distance within each realm.

**Extended Data Fig. 2:**
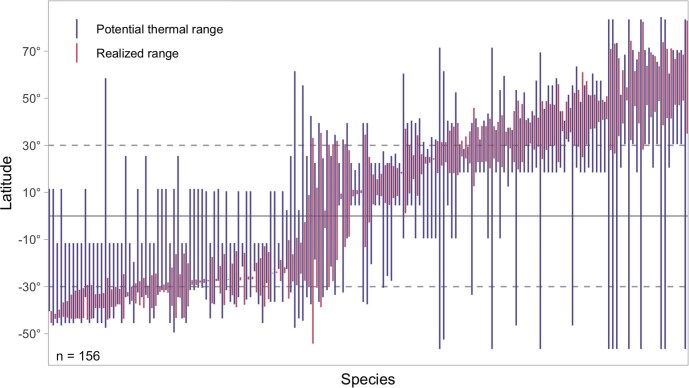
Latitudinal extent of realized and potential thermal ranges. Each vertical line represents the span across latitude of each species’ realized (pink) or potential thermal range (purple) for species in which we could measure range filling (n = 156 species). Realized ranges of species with ranges centred closer to the tropics (towards the centre of the x-axis) more completely fill their potential thermal ranges, while ranges of higher-latitude species tend to underfill latitudes towards the equator within their potential thermal ranges.

When niche underfilling was measured in terms of underfilled area rather than temperatures, results also were consistent with predictions from the temperate-trade-off hypothesis on land. Contrary to predictions from the reduced-abiotic-limitation-in-the-tropics hypothesis, total range filling did not change with the absolute latitude of a species range (Fig. 4a, Extended Data Fig. 1 and Extended Data Table 3). However, range underfilling in terrestrial species was generally biased towards species’ equatorward range edges and this bias was greater for species living at higher latitudes (Fig. 4b and red-blue colour scale, Extended Data Figs. 1 and 2 and Extended Data Table 3), as was predicted by the temperate-trade-off hypothesis. By contrast, although marine species underfilled much of their potential thermal ranges, indicating that they do not occupy all thermally tolerable areas, this range underfilling showed little latitudinal or thermal bias (white points and model fits close to zero; Fig. 4b, Extended Data Fig. 1 and Extended Data Table 3), although sample sizes were relatively small.

Unlike patterns of warm niche filling and geographic range filling, patterns of cool niche filling were not consistent with predictions from either hypothesis. Whereas the hypotheses predicted that species would either fill or underfill the cool extremes of their potential thermal niche (Fig. 1a), we instead found that thermal tolerance limits tended to underpredict species’ realized thermal niches (that is, species occupy places where temperatures appear to be colder than their cold tolerance limits; solid blue lines in Figs. 2 and 3). Underprediction signals imperfect assessment of the fundamental or realized thermal niche and we explore variation in niche underprediction as a means to understand the causes (for example, unmodelled microclimates and cold season dormancy). Cool niche underprediction increased with the absolute latitudinal midpoint of a species’ range in terrestrial and intertidal marine species (Figs. 2 and 3b,d), but not in subtidal marine species, in which cool niche filling was closer to zero (perfect filling) and did not change with latitude (Figs. 2 and 3f, Extended Data Fig. 1 and Extended Data Table 2). Nevertheless, this pattern suggests that species from all latitudes are filling rather than underfilling their cool thermal niche, so is more consistent with predictions from the temperate-trade-off hypothesis (Fig. 1a).

**Extended Data Fig. 3:**
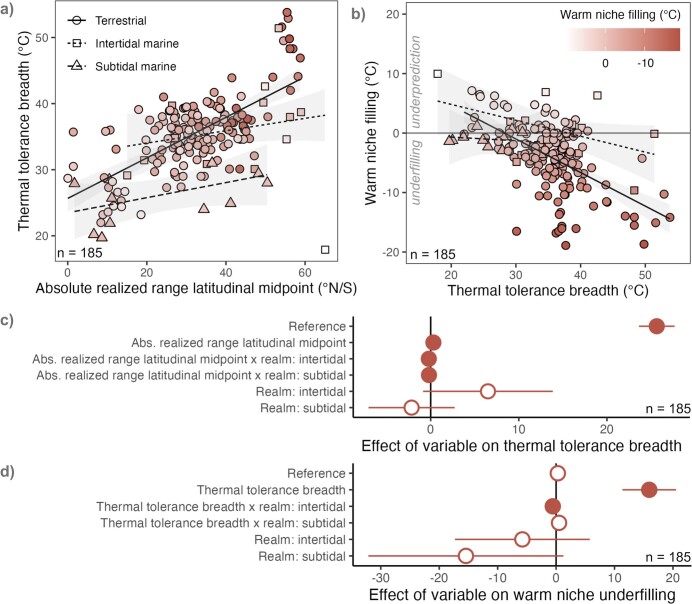
Thermal tolerance breadth increases with latitude and warm niche underfilling is greater in species with broader thermal tolerances on land. **a**, **c**. Linear models fit to the subset of species with both thermal tolerance limits (n = 185 species) indicate that thermal tolerance breath increases with the latitudinal midpoint of a species range on land (circles) and less so in the ocean (intertidal species: squares; subtidal species: triangles). **b**, **d**. Lines in panels a-b represent model predicted relationships with ribbons representing 95% confidence intervals (n = 185 species). Dots in panels c-d represent parameter estimates while lines represent associated 95% confidence intervals (n = 185 species). Solid dots distinguishing parameter estimates whose 95% confidence intervals do not overlap 0. Contrast coefficients in panels **c-d** are in reference to the terrestrial realm.

We thus find that patterns of thermal niche and area-based underfilling are strongly consistent with predictions from the temperate-trade-off hypothesis on land. In addition, the hypothesis assumes that thermal niche breadth increases with latitude and predicts that warm niche underfilling increases with thermal niche breadth. We found support for both relationships in terrestrial species in the subset of data for which both heat and cold tolerance limits were available (Extended Data Fig. 3 and Supplementary Table 1).

Although we expected factors other than latitude to explain variation in thermal niche and range filling, we found no relationship between either dispersal distance or body size and how well species filled their potential thermal niche or range (Extended Data Fig. 1 and Extended Data Tables 2 and 3). We did find that thermal niche filling was greater in species with larger geographic ranges (Extended Data Fig. 1 and Extended Data Table 2), consistent with the hypothesis that larger-ranged species are less ecologically specialized and thus more limited by temperature than by other ecological factors^40^. However, this finding could be considered as tautological if thermal breadths are globally constrained, as larger ranges would always take up a greater proportion of somewhat fixed thermal niche breadths.

**Extended Data Fig. 4:**
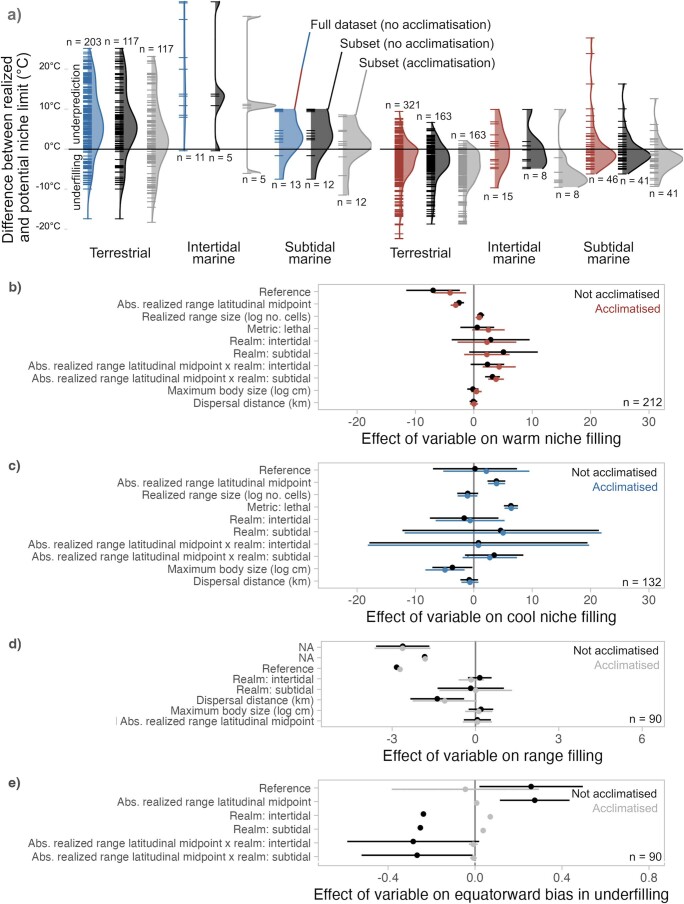
Effect of acclimatization on potential thermal niche filling and model results. **a**. Allowing plasticity of thermal limits via acclimatization to local temperatures using a subset of data (grey distributions) decreased cool and warm niche underprediction across all realms compared to when species thermal limits were assumed to not vary temporally or spatially across their range (coloured distribution: full dataset; black distributions: subset of full dataset; see Supplementary Methods – Section 5). The subset of species used in the acclimatization analysis represent species for which an acclimation response ratio could be estimated and for which the experimental acclimation temperature used in the thermal assay was known. All three distributions are shown to demonstrate that the shift in central tendency was due to the acclimatization correction applied to the subset and was not due to the subsetting of data itself. **b–e**. Dot-and-whisker plot comparing model-averaged results for models fit to (b) warm niche filling (n = 212 species), (c) cool niche filling (n = 132 species), (d) range filling (n = 90 species) and (e) equatorward bias in underfilling values (n = 90 species) for a subset of species when we simulated acclimatization to local temperatures (black) versus when the species’ thermal tolerance limits were assumed to remain constant across its range (coloured). Dots represent parameter estimates while lines represent associated 95% confidence intervals. Allowing fundamental thermal niche limits to acclimatize to local temperatures did not affect conclusions from model results. Although contrast coefficient estimates for the effect of absolute realized range latitudinal midpoint on equatorward bias in underfilling differed when acclimatization was applied to the subset (panel e), the trend across latitude within the subset without acclimatization was not representative of the trend across latitude within the full dataset.

**Extended Data Fig. 5:**
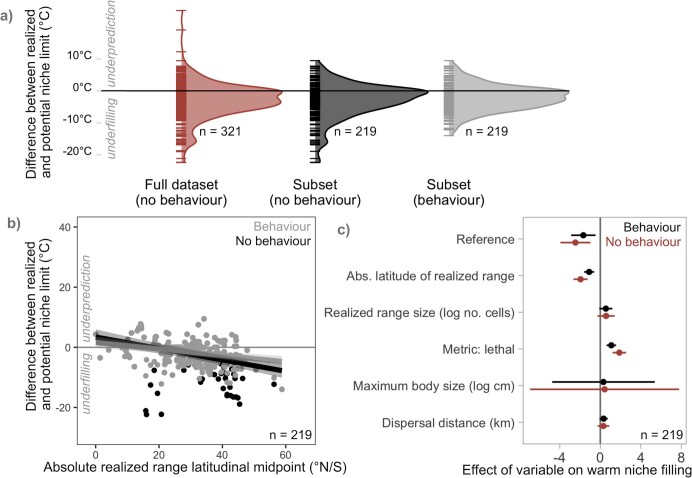
Effect of behavioural thermoregulation on warm potential thermal niche filling and model results on land. **a**. When we simulated the movement of terrestrial species between the sun and shade to maintain their preferred temperature (light grey distribution), warm niche underfilling decreased compared to when species were assumed to always remain in the shade (red distribution: full dataset; black distributions: subset of full dataset; see Supplementary Methods – Section 6). The subset represents terrestrial species for which thermal preference data were available from the literature. All three distributions are shown to demonstrate that the shift in central tendency was due to the behaviour correction applied to the subset and was not due to the subsetting of data itself. **b**. Linear mixed-effect models fit to the subset of terrestrial warm niche filling values (n = 219 species) with (grey) and without (black) behavioural thermoregulation show that allowing behaviour decreases the effect of latitude on warm thermal niche filling but the effect remains. **c**. Comparison of model-averaged results for a subset of terrestrial species (n = 219 species) allowed to behaviourally thermoregulate towards their preferred temperature (black) versus when they were assumed to always remain in the shade (red). Dots represent parameter estimates while lines represent associated 95% confidence intervals.

The observed latitudinal patterns of niche and range filling were generally robust to taxonomic non-independence and variation in thermal limit assay methodology and remained similar in magnitude after we incorporated phenotypic plasticity and thermoregulatory behaviour. We relaxed the assumption that species’ thermal tolerance limits are fixed over space and time by simulating acclimatization of species to seasonal temperatures across the landscape (Methods). This led to broader potential thermal niches on average and reduced the extent of both warm and cool niche underprediction (grey shadow compared to coloured density distributions in Fig. 3g,h and Extended Data Fig. 4a; warm niche underprediction on land reduced by ~10 °C, cool niche underprediction reduced by ~5 °C). However, the relationships with latitude did not change (Extended Data Fig. 4b–e and Supplementary Table 2). Similarly, simulating thermoregulatory behaviour in a subset of terrestrial species (*n* = 219) by relaxing the assumption that animals always prefer shaded habitat accounted for a portion of warm niche underfilling (Extended Data Fig. 5a and Supplementary Methods Section 6) but patterns across latitude remained (Extended Data Fig. 5b,c and Supplementary Table 3). Thus, although the assumptions made about phenotypic plasticity and thermoregulatory behaviour affect measurements of thermal niche filling, they do not affect the latitudinal patterns reported here.

**Extended Data Fig. 6:**
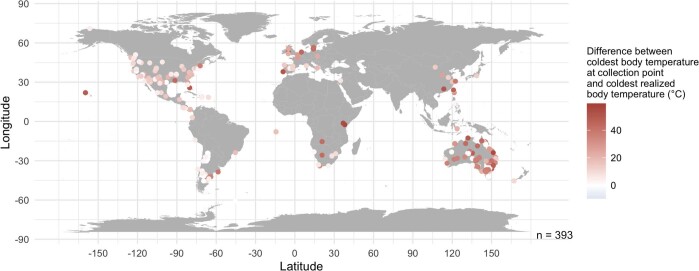
Map of collection location of organisms used in thermal assays. Points represent the geographic coordinates of the collection locations where wild organisms used to assay species thermal tolerance limits (fundamental thermal niche limits) were captured. Colour of points indicates the difference between the most extreme cool body temperature at the collection location and the most extreme cool body temperature across the species’ realized range. The abundance of red points indicates that collection locations of specimens were often from warmer parts of species’ ranges.

Even after simulating acclimatization and behaviour, some niche underprediction remained, which suggests error in assessing the fundamental or realized thermal niche. Remaining niche underprediction might be explained by organisms’ ability to vary their thermal limits via physiological plasticity that was not accounted for by our simulation (for example, rapid cold hardening^37^ and local adaptation of acclimation ability). Moreover, although we attempted to use only temperatures during the active periods of species with known seasonal dormancy, limited information on the timing and duration of dormancy might have led to underestimates that could falsely restrict the potential thermal niche (Supplementary Methods Section 7). It is interesting to consider why niche underprediction was biased toward the cool rather than warm edge of the thermal niche. Individuals in experimental trials were often collected from warmer parts of a species’ range (Extended Data Fig. 6), meaning our analysis might underestimate cold tolerance in colder parts of the range if assayed animals were locally adapted. Alternatively, the cold bias of niche underprediction might be caused by methodological error. Since physiological performance tends to decline more slowly as individuals reach their cold versus heat tolerance limits, one might expect greater error in the estimation of experimental endpoints at the cool versus warm edge of the fundamental thermal niche. Additionally, there is potentially a weak connection between laboratory-assayed cold tolerance and in situ survival in microhabitats because cooling rates experienced within winter burrows are typically much slower than those used in experiments^41^.

Under the interpretation that the increase in warm niche underfilling on land is linked to biotic interactions, it is intriguing to consider why marine species do not show the same pattern. The temperate-trade-off hypothesis assumes that higher-latitude species have broader thermal niches and are outperformed by lower latitude species with narrower thermal niches. However, thermal tolerance breadths of marine species increased only slightly with latitude in our data (Extended Data Fig. 3 and Supplementary Table 1) and globally have remarkably low variation over most latitudes^42^. Hence, under the temperate-trade-off hypothesis, little loss in performance is expected for marine species at higher latitudes. It is also possible that there is no clear latitudinal pattern of biotic exclusion in marine systems because species interaction intensity does not vary as consistently with latitude in the ocean^43^. Either way, whether driven by differences in latitudinal patterns of thermal tolerance breadth or species interaction intensity, empirical evidence that marine ranges are more responsive to climate change^3,39^ suggests that there is a biological mechanism behind the difference in warm underfilling in species on land versus in the ocean.

Although warm niche underfilling is possibly linked to biotic interactions, other mechanisms could be responsible for the observed patterns. First, a trade-off between cold adaptation and performance might exclude high-latitude species from warm environments irrespective of how it affects biotic interactions specifically (for example, if cold tolerance trades off with drought tolerance). Second, even without a trade-off, other abiotic niche requirements may be limiting in warm areas (for example, moisture in the hot desert belts, oxygen in warmer ocean regions, duration rather than extremeness of heat) and may act along longitudinally rather than latitudinally across a range. Third, warm underfilling might occur because species’ ecological limits to population growth are more limiting than an individual organisms’ capacity to function under heat stress (as generally measured in experiments). For example, temporal variability in temperatures and a history of thermal stress can reduce heat tolerance at the population scale^44–46^, resulting in mismatch between individual acute thermal tolerance measured in the laboratory and the thermal limits of long-term population survival. Similarly, if early life stages are more heat-sensitive than the adults typically assayed (for example, refs. ^42,47^) or if sublethal temperatures limit critical life-history functions (for example, mate-finding and gamete viability), populations might not be able to persist in temperatures that can be tolerated by adult organisms. Lastly, our analyses might have overestimated warm niche underfilling across all latitudes by assuming that terrestrial species can exploit shaded microhabitats. If land animals are unable to seek shade^48^, species might be in greater thermal danger than patterns here suggest (see ref. ^49^). Distinguishing among the possible mechanisms of warm niche underfilling is important to understand species’ temperature sensitivities under climate warming (Supplementary Discussion Section 2).

Observational evidence of variation in species’ range shifts in response to climate warming already indicates greater sensitivities in marine compared to terrestrial species^3,39^, consistent with the finding that marine species more closely fill their thermal niches. Observed range shifts can be used to test additional hypotheses stemming from results presented here; namely, if thermal niche underfilling is associated with lower sensitivity to temperature changes, we predict marine species and species in the terrestrial tropics to be more sensitive to temperature change. We also predict warm range edges of extratropical terrestrial species to be less sensitive to temperature change than cold range edges, with contractions more likely to be tied to drought or climate-related increases of antagonistically interacting species. Our results show that general patterns of temperature limitation among species emerge despite the existence of the many factors and their complex interactions that shape species distributions. The shared evolutionary history of all lifeforms might likewise lead to general patterns in how biodiversity and ecosystem functions respond to contemporary climate change.

## Methods

### Fundamental thermal niches

We defined the fundamental thermal niche as the range of temperatures within experimental estimates of a species’ upper and lower thermal tolerance limits (following refs. ^36,37^; see Supplementary Methods Section 1 for formal definition). Thermal tolerance limits are measured in a laboratory in the absence of other limiting factors and are derived independently from a species’ current observed distribution, thus they provide an estimate of the fundamental thermal niche. We retrieved thermal tolerance data from the supplementary material of ref. ^50^, which represents a curated subset of the Globtherm database^51^ containing a single estimate per species of a critical limit (the body temperature at which an organism loses the ability to perform a critical function) or lethal limit (the body temperature at which an organism dies) at upper and/or lower temperature extremes. We subset data to include only subtidal marine, intertidal marine and terrestrial ectothermic animal species, deciding to exclude species from freshwater habitats (*n* = 118) since we lack appropriate freshwater temperature data at the global scale. We included terrestrial species with a freshwater larval stage since thermal limit assays were performed on adult organisms. This yielded a dataset of 870 species with estimates of either one or both limits of the fundamental thermal niche.

### Realized ranges and traits

For each species for which we had one or both limits of the fundamental thermal niche, we extracted geographic range maps in the form of polygons (extents of occurrence) from two sources: the International Union for the Conservation of Nature (IUCN) spatial data portal^52^ (*n* = 318) and the global assessment of reptile distributions data repository^53^ (*n* = 51). Additional range maps were inferred by fitting convex hulls to carefully filtered occurrence records obtained from the Global Biodiversity Information Facility (gbif.org/occurrence/search; accessed December 31, 2016)^54^ (*n* = 225) following IUCN methods (iucnredlist.org/resources/mappingstandards; accessed June 23, 2022; Supplementary Methods Section 2). When multiple range maps from different sources were available for a species, we used the IUCN range map in analyses (although results were not sensitive to the source of the realized range used; Supplementary Fig. 1). Although range map polygons are known to overpredict species distributions^55^, they were considered sufficient for the purposes of this analysis since our intent was to measure the temperatures across species ranges and environmental conditions measured across range polygons are highly correlated to those measured using finer-scale species occurrence data^56^. Of the 870 non-freshwater ectothermic animal species in the Globtherm database, we were able to procure range maps for 474 of them.

We converted each species range map polygon to 1° × 1° resolution presence/absence grid and carried out the remainder of analyses at this spatial resolution. We chose to represent species distributions and the temperatures across them at this relatively coarse scale (~104 km^2^ grid cells) to capture the uncertainty in the location of species range edges that is caused by imperfect sampling and the dynamic nature of species distributions. For each species, we then searched the literature for a suite of traits chosen in accordance with our additional hypotheses (Extended Data Table 1 and Supplementary Methods Section 4) to include as predictor variables in models.

### Estimating body temperatures

We used climate data to estimate the warmest and coolest daily extreme body temperature each species would experience within each grid cell. For subtidal and intertidal marine species, we used coarse-grained macroclimatic data to characterize the span of daily body temperatures a species would experience across the globe. For subtidal species, we characterized body temperatures using sea surface temperature. Since intertidal species often experience both wet and dry body temperatures, we used both air and sea surface temperatures to define their thermal niches, selecting the more extreme of the two in coastal grid cells that contained both land and ocean. For grid cells in the ocean, we obtained daily maximum and minimum mean sea surface temperature climatologies over the period 1982–2020 from the NOAA optimum interpolation sea surface temperature v.2 high resolution dataset (psl.noaa.gov/data/gridded/data.noaa.oisst.v2.highres.html; accessed 15 June 2021) and converted the higher resolution grid to 1° × 1° resolution by aggregating cells and selecting the maximum or minimum value of aggregates. In land grid cells along the coast, we calculated daily maximum and minimum air surface temperature climatologies at a 1° × 1° grid resolution over the period 1950–2000 from Berkeley Earth land datasets (berkeleyearth.org/data/; accessed 14 October 2020).

Temperature estimates from coarse-grained macroclimatic data poorly represent the temperatures body terrestrial organisms might experience in microclimates^57^. To better characterize the extreme body temperatures of terrestrial organisms, we used species traits and simulated microclimatic data from NicheMapR^58^ to estimate species-specific hourly operative temperature climatologies in the sun and shade in each 1° × 1° grid cell. NicheMapR uses climate observations from weather stations and information on landscape features to model how weather conditions interact with local habitat to generate different microclimatic conditions. In the centre of each 1° × 1° grid cell, we used simulated environmental variables from NicheMapR (solar radiation, air temperature, soil surface temperature, wind velocity, relative humidity and wind velocity) combined with species trait data to model the equilibrium temperature of each terrestrial animal in its environment given heat exchanged via absorption and emission of radiation, convective heat dissipation and cooling due to evaporative water loss from the skin (Supplementary Methods Section 3). We were unable to model operative temperatures for 14 of the 401 terrestrial species because body size estimates were unavailable.

**Extended Data Fig. 7:**
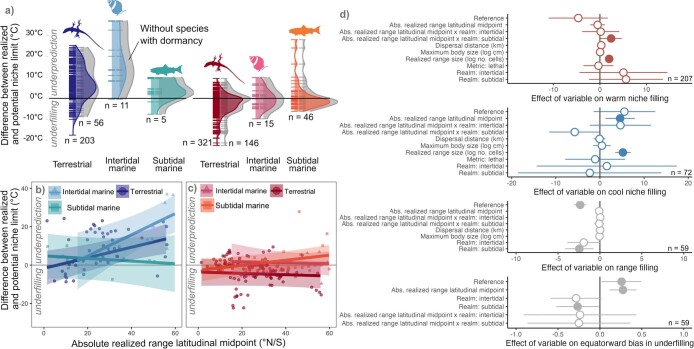
Effect of correction for dormancy on niche filling values and model results. Our main analysis corrected the body temperatures of species with seasonal dormancy to reflect only temperatures experienced during active periods. To ensure this correction did not substantially affect results, we repeated all analyses on the subset of species that, according to information available in the literature, are not known to undergo seasonal dormancy. **a**. We found that distributions of niche range filling values for the entire dataset (coloured) and the subset of species that do not undergo dormancy (grey) are similar. **b-d**. Linear mixed-effect models fit to the subset of species without seasonal dormancy also show similar results to models fit to the entire dataset (warm niche filling: n = 207 species; cool niche filling: n = 72 species; range filling and equatorward bias: n = 59 species). The increase of warm niche underfilling with latitude in terrestrial species was reduced when only non-dormant species were analysed (c), however this likely has to do with the removal of many high-latitude data points (as high-latitude species more often undergo seasonal dormancy). Lines and ribbons in panels b and c represent model predicted relationships and associated 95% confidence intervals. Dots and lines in panel d represent parameter estimates and associated 95% confidence intervals, with solid dots distinguishing estimates whose 95% confidence intervals do not overlap 0.

Estimating the operative temperature of an animal requires making assumptions about the microhabitat that individuals choose. As a reasonable first approximation of thermoregulation under extreme conditions, we assumed in our main analysis that species’ body temperatures would be equilibrated to the shade when experiencing hot extremes and to the sun when experiencing cold extremes. To do this, we defined the species’ extreme body temperatures using the hottest hourly shaded operative temperature and the coldest hourly exposed operative temperature. For species with seasonal dormancy, as informed by the literature, we masked body temperatures experienced during the six hottest and/or coldest months of the year before selecting the hottest and coldest extremes (Supplementary Methods Section 7) and tested our results for sensitivity to this method (Extended Data Fig. 7). We validated our operative temperature estimates and assumptions about microhabitat use using a dataset of empirical estimates of species’ field body temperatures^59^. The empirical field body temperatures of most species fell within the range of simulated operative temperatures at locations where field specimens were sampled; Supplementary Fig. 2. We additionally tested the sensitivity of operative temperature estimates to variation in model parameter values (Supplementary Fig. 3 and Supplementary Methods Section 3).

In summary, this process left us with two global grids of extreme body temperatures for each species: one representing the extreme warm body temperatures and the other representing the extreme cool body temperatures expected to be experienced by the species. For subtidal marine species, these extreme body temperatures were characterized by the average sea surface temperatures on the hottest or coldest day of the year. This was the same for intertidal species, except in coastal grid cells where temperatures might instead represent the average hottest or coldest air surface temperature on the hottest or coldest day of the year (if it was more extreme). Finally, for terrestrial species, temperatures represented the average hottest or coldest modelled hourly operative temperature of the animal in a reasonable refugial microhabitat on the hottest or coldest day of the year during the animal’s period of activity. Although we were not able to account for hourly temperature variation or opportunities for microhabitat use in marine settings, we opted to keep this level of detail in terrestrial settings where hourly temperature variation and microhabitat variation are greater and therefore more necessary to consider when approximating experienced temperatures.

### Potential and realized thermal niches

For each species, we first inferred the potential thermal niche in the form of a 1° × 1° resolution presence/absence grid by applying a series of species-specific restrictions to reduce available habitat. First, to avoid overestimating the potential range by including habitat in uninhabitable environments (for example, including areas of ocean in the potential niche of a terrestrial reptile) or habitat that is uninhabited due to large-scale, historical dispersal barriers (for example, continental divides), we restricted habitat to include only cells in the species’ inhabited realm (marine species, ocean cells; intertidal species, ocean cells within 200 km of the coastline and land within a 1° grid cell of the coastline; terrestrial species, cells within the biogeographic realm(s) contiguous with the species’ realized range, as determined by ref. ^60^). To restrict habitat by the species’ fundamental thermal niche, we then removed remaining grid cells where the species’ extreme body temperatures were hotter or colder (or both) than the species’ fundamental thermal niche limits. For species with only one available thermal tolerance limit, we used only one fundamental thermal niche limit to restrict the available habitat. We then used global grids of elevation (earthenv.org/topography) and depth (gebco.net/data_and_products/gridded_bathymetry_data/) to remove remaining cells of uninhabitable depth (marine) or altitude (terrestrial) when information on the species depth or elevational distributions was available in the literature (that is, known elevation or depth range, whether a marine species is pelagic or benthic-associated; Supplementary Methods Section 4). We applied the same depth and elevation correction to the realized range polygon of each species and, when information on depth or elevation distribution was unavailable (*n* = 175), we left the thermal niches as-is. We found that, overall, both niche underprediction and niche underfilling were reduced in species with depth or elevation-corrected thermal niches.

We then derived the potential and realized thermal niches in environmental space from estimates of the potential and realized range. To do this, we quantified the span (maximum and minimum) of extreme body temperatures occurring across the 1° × 1° resolution potential and realized presence/absence grids. For species with only one available fundamental thermal niche limit (upper limit only, *n* = 219; lower limit only, *n* = 44), the potential thermal niche was inferred from only one limit, which assumes that the potential range is not further constrained by the other thermal tolerance limit. Since we were unable to model operative temperatures for 14 terrestrial species who lacked body size estimates, we estimated a total of 460 potential thermal niches.

### Measuring thermal niche filling

In environmental space, we calculated warm and cool filling of the potential thermal niche as the difference between the potential and realized warm and cool thermal niche extremes, respectively. This metric is in °C. Negative values indicate that a species is underfilling its cool or warm potential thermal niche limit, whereas positive values indicate that thermal tolerance limits underpredict the realized thermal niche limit. A value of zero indicates that the species’ perfectly fills its thermal niche limit. Some Antarctic and island specialist species did not have temperature data available across their realized range (*n* = 24), allowing us to measure niche filling in thermal space for only 436 species. These species were mostly reptiles (*n* = 278), with the remainder being amphibians (*n* = 60), fish (*n* = 26) and arthropods, molluscs or marine invertebrates (*n* = 72). For species with available body temperature data and both warm and cool thermal limits (*n* = 185), we calculated both warm and cool thermal niche filling, while only one niche filling value was calculated for species with only one thermal limit (warm niche filling only, *n* = 206; cool niche filling only, *n* = 44).

For species with potential thermal niches inferred using both fundamental limits (*n* = 185), we also calculated filling of the potential thermal niche in geographic space (range filling). We calculated range filling as the proportion of cells in the species’ potential thermal range that the species occupies, which ignores areas of geographic underprediction. While in environmental space a niche filling value of 0 indicates perfect niche filling, in geographic space a range filling value of 0 indicates complete range underfilling (species occupies no areas of its potential range) and a value of 1 indicates perfect range filling (that is, species occupies all areas of its potential range). We also analysed the equatorward bias in range underfilling by calculating the difference between the proportion of the potential thermal range that is underfilled in the equatorward and poleward range halves. To do this, we split the potential range in half latitudinally at the midlatitude of occupied cells, calculated range underfilling for either half (that is, the proportion of cells in either half of the potential thermal range that were not occupied) and subtracted range underfilling in the equatorward half from underfilling in the poleward half. We did not analyse range filling or equatorward bias in underfilling for species whose realized range and potential thermal range did not overlap (*n* = 37), leaving range filling estimates for 160 species.

### Analyses

To test our hypotheses, we fit linear mixed-effect models to warm niche filling, cool niche filling, range filling and equatorward bias in underfilling separately using the nlme package^61^. We included the following traits as fixed effects: realm (categorical); absolute latitudinal midpoint of realized range (continuous); dispersal distance (continuous); log(maximum body size) (continuous); and log(realized range size) (continuous). We excluded log(realized range size) from the range filling model as we recognized that shared geometric constraints imposed by continental barriers on potential ranges might result in a circularity between range size and geographic range filling (Supplementary Discussion Section 1). Because we expected the relationship between niche filling and latitude to differ across realms on the basis of previous findings^37^, we included an interaction term between realm and absolute latitudinal midpoint of the realized range. We only modelled species for which all traits were known, which slightly reduced our sample sizes (warm niche filling, *n* = 382; cool niche filling, *n* = 227; range filling and equatorward bias in range filling, *n* = 156). A final list and taxonomic breakdown of species included in the models is presented in Supplementary Tables 6 and 7. Because it was a proportion of area, we log-transformed range-filling values before modelling. We checked for collinearity between fixed effects using variance inflation factors and removed one of the collinear variables when the factor was greater than three^62^. For species with both thermal tolerance limits (*n* = 185), we also fit linear models to thermal niche breadth (that is, the difference in °C between the maximum and minimum thermal tolerance limits) as a function of absolute latitudinal midpoint of the realized range and warm niche filling as a function of thermal tolerance breadth, allowing the slope and intercept to vary between realms.

Combining data from diverse sources and taxa can introduce non-independence that can be accounted for through a modelling approach. To account for consistent differences caused by different experimental thermal limit testing procedures, we included thermal limit metric type (critical or lethal) as a fixed effect in our niche filling models. This was not possible for the range filling model as metric type was found to be collinear with the term ‘realm’. To account for non-independence of data due to shared evolutionary history and due to methodological differences in measuring the fundamental thermal niche across taxonomic groups, taxonomic categories from class through to the species level were included in all models as nested random effects on the intercept.

We ensured that this modelling framework sufficiently controlled for phylogenetic non-independence by comparing results to those estimated in a phylogenetic generalized least squares (PGLS) analysis^63^. Using a time-calibrated phylogeny^64^, we estimated a variance–covariance matrix describing the shared evolutionary history between species in our data for which divergence times were available (*n* = 376). We used this matrix to run PGLS models using the gls function of the nlme package^61^, including the same fixed effects as in our linear mixed-effect models. We re-ran our linear mixed-effect models on the subset of data for which phylogenetic information was available to allow comparison of the results obtained using the two methods. We found that within the data subsets, fixed effect coefficients estimated by both methods did not differ substantially (Supplementary Fig. 4). We present the main analysis using estimates from the linear mixed-effect modelling approach since it allows us to include a larger sample size of data.

We performed model averaging rather than selecting a single top model to avoid introducing uncertainty through the model selection process. We used the MuMIn package^65^ to run all candidate models, which included all possible combinations of terms. We performed multimodel averaging with maximum likelihood estimation to identify the confidence set or the models comprising the top 95% of model weight. We report full averages of coefficients. To ensure that the assumption of normality was met, model residuals were visually inspected. To ensure that the exclusion of certain explanatory variables from some models did not dramatically affect the estimation of other model parameters (one possible problem introduced by model averaging, ref. ^66^), parameter estimates from models in the confidence set were visually compared to each other and to the model average (Supplementary Fig. 5).

### Acclimatization sensitivity analysis

While our main analysis assumed that a single upper and lower thermal tolerance limit defines a species’ fundamental thermal niche across its range, we used reduced datasets (warm niche filling, *n* = 212; cool niche filling, *n* = 134; range filling, *n* = 90) to simulate the potential plasticity of fundamental thermal limits to local temperatures. We compiled data on acclimation response ratios (ARRs) or the slope of a linear regression fit to upper or lower thermal limits as a function of experimental acclimation temperature, from published^67–70^ databases and augmented these with individual studies using a new literature search (Supplementary Fig. 6a). We averaged ARRs within species and used these to simulate the acclimatized fundamental thermal niche limits in each grid cell for each species in our study. In each cell, we used the ARR to calculate what the upper thermal limit would be if acclimatized to the maximum temperature occurring within 7 days before the hottest day and what the lower thermal limit would be if acclimatized to the minimum temperature occurring within 7 weeks before the coldest day (based on information available on the time course of acclimatization of upper and lower limits^71–73^; Supplementary Fig. 6b). For species in our data with no available species-specific ARR estimate, we used a class-averaged or realm-averaged ARR (see Supplementary Methods Section 5 for full description of analysis).

We inferred species’ acclimatized potential thermal niches using the grid cell-specific acclimatized fundamental thermal niche limits. We did this by comparing a species’ extreme body temperatures in a grid cell to its acclimatized thermal tolerance limits in that grid cell and retaining only cells where extreme body temperatures fell within the acclimatized fundamental thermal niche limits. We then modelled acclimatized range filling and warm and cool thermal niche filling using the same linear mixed-effect modelling framework as in our main analysis and additionally fit our original models to only species included in the acclimation subset (Extended Data Fig. 4 and Supplementary Tables 2 and 4).

### Behavioural thermoregulation

While our main analysis assumed that terrestrial animals prefer to remain in shaded habitat during the hottest hour on the hottest day of the year, this might not be true; in colder places, animals might remain in the sun to maintain a warmer body temperature, even when experiencing the hottest yearly temperature extreme. To ensure this assumption about thermoregulatory behaviour did not affect our results, we gathered estimates of terrestrial species’ preferred body temperature from the literature (defined as the body temperatures that species maintain in nature^74^, either estimated from measurements of preferred temperature in an experimental thermal gradient (*T*_pref_) or from measurements of field body temperature (*T*_b_)). For the subset of terrestrial species (*n* = 219) for which a preferred temperature estimate was available, we then adjusted realized upper thermal niche limits to reflect the hottest temperature the species would experience across its realized range if it is assumed to behaviourally thermoregulate towards its weighted mean preferred temperature by moving between the sun and shade (Supplementary Methods Section 6). We then modelled warm thermal niche filling in this subset using the same linear mixed-effect modelling framework as in our main analysis and fit our original model of warm thermal niche filling to only species included in the behaviour subset (Extended Data Fig. 6 and Supplementary Table 3). Although marine species can also regulate their body temperature by moving to different depths of the water column, we could not carry out a similar analysis for marine species due to the limitations imposed by our small sample size.

### Reporting summary

Further information on research design is available in the Nature Portfolio Reporting Summary linked to this article.

### Supplementary information

Supplementary Information Supplementary Methods, Discussion, References, Figs. 1–6 and Tables 1–7.

Reporting Summary

## Figures and Tables

**Extended Data Table 1:**
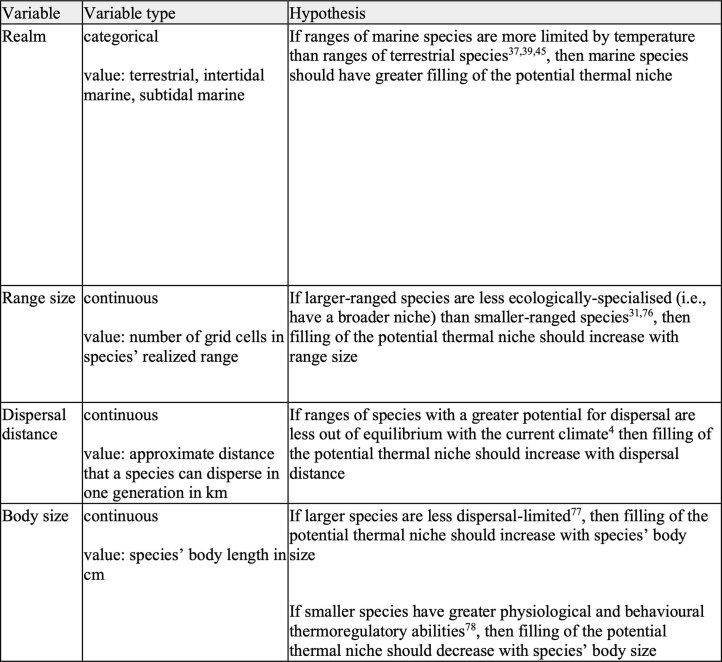
Additional hypotheses for how filling of the potential thermal niche varies according to latitude, realm and range size and traits

**Extended Data Table 2:**
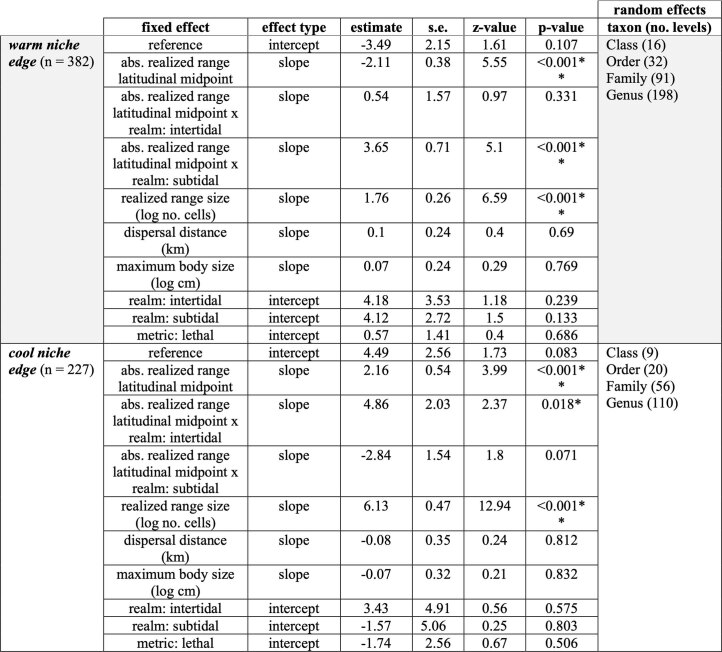
Average model summaries for the confidence set of models of warm and cool potential thermal niche filling as a function of species traits

Reference levels for contrast coefficients and diagnostics (*z*- and *p*-values) of fixed effects were terrestrial for realm and critical for thermal limit metric. A single asterisk (*) denotes a *p*-value less than 0.05 and double (**) signals a *p*-value less than 0.01.

**Extended Data Table 3:**
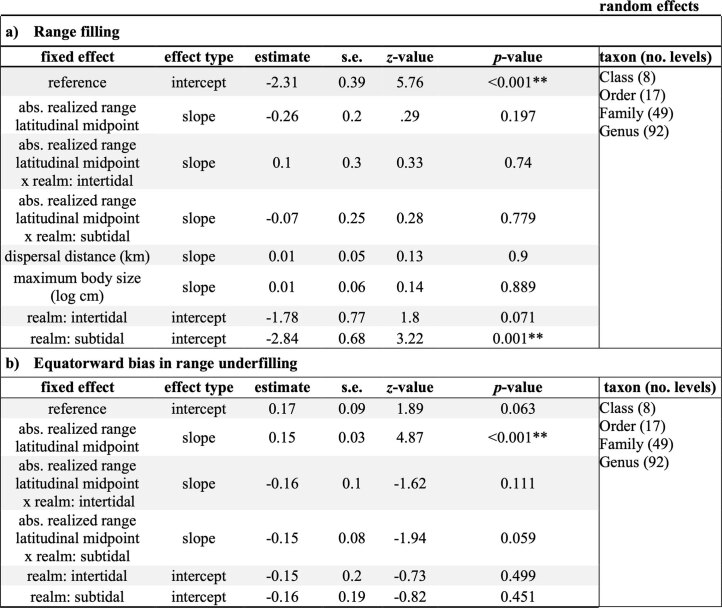
Average model summaries for the confidence set of models of range filling and equatorward bias in range underfilling as a function of species traits

The reference level for contrast coefficients and diagnostics (z- and p-values) of the fixed effect realm was terrestrial. A single asterisk (*) denotes a p-value less than 0.05 and double (**) signals a p-value less than 0.01 (n = 156 species).

## Data Availability

A minimum dataset needed to reproduce the results presented in this analysis can be found in the figshare repository associated with this article^75^. This repository also includes an archived version of the Github repository, which contains initial and intermediate data files, as well as a folder of large files that exceed the GitHub storage limit.

## Code availability

All code needed to reproduce analyses presented in the article is available in an archived Github repository^76^.
