# Supplementary material for: Temperate species underfill their tropical thermal potentials on land
Source: Nat Ecol Evol. 2023 Nov 6;7(12):1993–2003. doi: 10.1038/s41559-023-02239-x (PMC10697837; doi:10.1038/s41559-023-02239-x)
Supplement: Supplementary file 1 — Supplementary Methods, Discussion, References, Figs. 1–6 and Tables 1–7. [file 41559_2023_2239_MOESM1_ESM.pdf]

nature ecology & evolution

Article

https://doi.org/10.1038/s41559-023-02239-x

Temperate species underfill their tropical thermal potentials on land

In the format provided by the authors and unedited

Table of Contents

Supplementary Methods
Section 1. Technical definition of the fundamental, potential, and realized thermal niche… 2
Section 2. Inferring range polygons from GBIF occurrence data…………………………..…. 2
Section 3. Simulating operative body temperatures for terrestrial species…………………. 3
Section 4. Collation and use of species traits and restriction of potential thermal niche by depth and latitude……………………………………………………………………5
Section 5. Plasticity of thermal limits……………………………………………………. 6
Section 6. Behavioural thermoregulation on land…………………………………………7
Section 7. Accounting for seasonal dormancy……………………………………………8

Supplementary Discussion
Section 1. Relationship between range filling and realized range size……………………10
Section 2. Drivers of niche underfilling and climate sensitivity………………………….10

References……………………………………………………………………………12

Supplementary Figures
Fig S1……………………………………………………………………………14
Fig S2……………………………………………………………………………15
Fig S3……………………………………………………………………………16
Fig S4……………………………………………………………………………17
Fig S5……………………………………………………………………………18
Fig S6……………………………………………………………………………19

Supplementary Tables
Table S1…………………………………………………………………………20
Table S2…………………………………………………………………………21
Table S3…………………………………………………………………………22
Table S4…………………………………………………………………………23
Table S5…………………………………………………………………………24
Table S6…………………………………………………………………………25
Table S7…………………………………………………………………………35

Supplementary Methods

Section 1. Technical definition of the fundamental, potential, and realized thermal niche

Below, we use symbols from Soberon & Arroyo-Peña[1](2017) to define the fundamental, realized, and potential thermal niche in our study.

Fundamental thermal niche. The fundamental thermal niche (N_F) is defined as the closed range of temperatures between a species lower (T_{min}) and upper (T_{max}) critical or lethal thermal tolerance limits (with thermal tolerance limits from Sunday et al.[2]).

Encounterable temperatures. Temperatures in the current environment across a geographic area, G, at time t can be described as E(t, G). Since a species might not be able to access habitat that is far away from its current range, we define the encounterable geographic region, G, as the habitat within the biogeographic realm a species currently occupies.

Potential thermal niche. The intersection of the fundamental thermal niche N_F with the accessible environment E(t, G) defines the potential thermal niche, N*(t, G).

Realized thermal niche. The realized thermal niche N_R(t, G) was estimated as the closed range of temperatures within E(t, G) falling within the species' realized range (estimated using an IUCN Extent of Occurrence map or by fitting a convex hull to filtered GBIF occurrence records).

Section 2. Inferring range polygons from GBIF occurrence data

To infer range polygons from Global Biodiversity Information Facility (GBIF)[3] occurrence data, we first extensively filtered the occurrence records. GBIF data are biased toward highly populated/travelled areas and countries with a strong history of European taxonomy (Europe, North America, Australia, New Zealand, South Africa; see map on GBIF.org homepage), resulting in poor spatial resolution for species range edges that fall in under-represented areas (e.g., equatorial Africa, Russia, northern Canada). Therefore, before inferring realized ranges, we filtered GBIF point occurrences through comparison to independently described native ranges, found by searching the primary published literature (Web of Science), online sources and databases (e.g., eFloras), and reference books available *via* the University of British Columbia library[4]. If a reliable source confirming the northern and southern range limits was unavailable or if a species had fewer than 30 occurrence observations, the species was discarded.

When an independent range description was available, we compared the GBIF point occurrences to it and took one of three actions: 1) discarded the species if the GBIF occurrences were very different from the described range (e.g., if it appeared multiple species were combined due to identification errors, if occurrence records from the northern or southern range edge were missing); 2) kept the GBIF occurrences without alteration if they matched the independent range description; or 3) used the range description to remove outlier occurrence records from outside the range. We then inferred range polygons following the IUCN protocol (iucnredlist.org/resources/mappingstandards) by fitting convex hulls around the filtered occurrence records for all species retained after the filtering process (n = 225) and created a smoothed polygon using Bezier Interpolation. Smoothed polygons were then clipped considering

the realm of the species (subtidal marine, intertidal marine or terrestrial) using polygons of recognized ecoregions[5,6].

Section 3. Simulating operative body temperatures for terrestrial species

To estimate the maximum and minimum temperatures that terrestrial species could experience in their habitats, we modelled the operative body temperatures (T_e, ºC) of each species in its environment, factoring in both the habitat available for thermoregulation and thermal constraints on activity (e.g., refs [7–9]).

In each 1° × 1° grid cell, we used NicheMapR[10] to simulate environmental variables (solar radiation, air temperature, soil surface temperature, wind velocity, relative humidity, and wind velocity) at 1 cm from the ground in both sun-exposed and shaded conditions (90% of shade) in the center of the cell. We used these environmental variables to model operative body temperatures (T_e, ºC) of terrestrial ectotherms as the equilibrium temperature of the animal in its environment given heat exchanged via absorption and emission of radiation, convective heat dissipation, and the role of cooling due to evaporative water loss from the skin[11,12].

$$T_e = T_a + \frac{R_{sol} + R_{lw} - \epsilon\sigma(T_a + 273)^4 - \lambda E}{h_R + h_c} \tag{1}$$

T_a is air temperature (ºC), and R_{sol} and R_{lw} are absorbed short- and long-wave radiation (W). The term $\varepsilon\sigma(T_a + 273)^4$ denotes heat dissipated via thermal radiation, where ε is the infrared emissivity of the body (set here to 0.965)[12] and σ is the Stefan-Boltzmann constant (5.67×10^{-8} $Wm^{-2}K^{-4}$). The coefficients h_R and h_c are, respectively, radiative and conductive heat transfer coefficients, estimated here as $h_R = 4\epsilon\sigma(T_a + 273)^3$and $h_c = c_p\left(1.4 + 0.135\sqrt{\frac{v}{d}}\right)$ where c_p is the specific heat of air (29.3 $Jmol^{-1}{}^{o}C^{-1}$), v is wind speed (ms^{-1}), and d is the characteristic length of the body (m)[11]. Finally, λE represents evaporative cooling, where λ is latent heat of water (2257 Jg^{-1}), and E is evaporative water loss from the skin ($g\ s^{-1}$). We estimated evaporative water loss from the skin for both dry- and wet-skinned ectotherms using the equation $E = \frac{e_s - e_a}{R_{tot}}$ [13], e_s - e_a is the gradient of water vapour density between the animal and the surrounding air (gm^{-3}) at air temperature (e_a = relative humidity × e_s), and R_{tot} is the total resistance to water loss (sm^{-1}). We used different values for R_{tot} to simulate either amphibians (300 sm^{-1})[13] or dry-skinned species such as reptiles and insects (6×10^5 sm^{-1})[14].

To model short- and long-wave absorbed radiation (R_{sol} and R_{lw}), we used the expressions $R_{sol} = \alpha_s F_a S$ and $R_{lw} = \alpha_{lw}(F_a L_a + F_g L_g)$, where α_s and α_{lw} represent skin absorptances to short- and long-wave radiation, set here to 0.9 and 0.965, respectively, following Buckley (2008)[12]. The factors F_a and F_g represent the proportion of skin surface area receiving incoming radiation from the sky and from the ground, respectively, and were both set to 0.5[12]. Finally, S represents total incoming short-wave radiation from the sun (Wm^{-2}), $L_a = 9.2 \times 10^{-6}\,\sigma\,(T_a + 273)^6$ is long-wave radiation coming from the sky[12], and $L_g = 0.965\,\sigma\,(T_g + 273)^4$, where T_g is soil surface temperature (ºC), is long-wave radiation from the ground[11,12].

Thoroughly validating T_e estimates requires measuring the temperature of animal replicas when placed in the sun and shade in different locations (see, for example, ref [15]).

Because this is not possible at the global scale, we used a pre-existing dataset of lizard species' field body temperatures[16] to ensure our modeled T_e estimates were reasonable approximations of the body temperatures species experience in their habitat. Since lizards move between heterogeneous thermal habitat to maintain their optimum body temperature (e.g., refs [17,18]), the mean field body temperature (T_b) of a lizard should fall within the range of possible T_e modelled at the location where T_b was assayed. For lizard species in our dataset with at least one T_b estimate assayed at a known collection location (from Algar et al. 2018[16], n = 102), we compared the reported T_b to the distribution of modeled mean hourly T_e across the year in the 1° x 1° grid cell containing the collection location. We found that T_b usually remains within the distribution of modeled T_e estimates at the collection location (n = 93, fig. S2a). The few cases where T_b was hotter than the hottest T_e at the collection location (n = 9) might be cases where T_b was measured during an extreme temperature event. Importantly, the cases where T_b was hotter than the hottest T_e at the collection location were not clustered to any specific latitude (fig. S2b). We also found that the difference between T_b and the coldest T_e increased with latitude (fig. S2b), likely because lizards spend more time in the sun at higher latitudes (a result that was also supported by our simulation of behavioral thermoregulation; Extended Data fig. 6).

Additionally, we conducted an analysis to test whether T_e estimates were sensitive to assumptions about habitat characteristics, species' burrow depth in the soil, and the properties of species' skin. Our main analysis assumed that soil type and soil reflectance do not vary across habitats (soil type = loam, soil reflectance = 0.15), that species' skin absorptance and emissivity are the same across species (α_{lw} = 0.965, α_s = 0.9, $\epsilon = 0.965$), and that species do not burrow (soil depth = 0cm). To test the sensitivity of results to these assumptions, we again used NicheMapR to simulate environmental variables in both the sun and shade, this time varying combinations of soil type (rock, sand, loam), soil reflectance (25%, 50%, 75%), and burrow depths (0cm, 10cm, 20cm, 30cm, 40cm, 50cm). For the first five species in our dataset, we calculated the maximum and minimum T_e of the species in each grid cell for every combination of soil type, soil reflectance, and burrow depth, allowing skin absorptance and emissivity to vary randomly within a reasonable range (short wave radiation skin absorptance parameter, α_s: 0.85 to 0.95[19]; infrared emissivity of the body, ϵ: 0.95 to 1.0[12]; long wave skin absorptance parameter, α_{lw}: equal to ϵ, since long wave absorptance in a given waveband is approximately equal to emissivity in that waveband[12]). For each set of conditions, we randomly sampled 100 skin absorptance and emissivity values, estimated the species' hourly T_e in the sun and shade for each, and recorded the maximum and minimum hourly T_e in the shade. This left us with a maximum and minimum hourly T_e in each grid cell for each of the five species, given variation in soil type, soil reflectance, burrow depth, skin absorptance, and skin emissivity, which we then compared to our original mean and range hourly T_e where these parameters were held constant.

We found that while allowing soil type, soil reflectance, burrow depth, and species skin properties to vary did not have a huge effect on the distribution of the species' cool operative temperatures, it had a small effect on the distribution of warm operative temperatures (fig. S3). The warm operative temperature distribution shifted towards warmer temperatures, indicating that allowing these model parameters to vary slightly reduces warm niche underfilling. This indicates that, as expected, variation in temperatures across microhabitats introduces some uncertainty into our estimates of warm niche underfilling.

Section 4. Collation and use of species traits and restriction of potential thermal niche by depth and latitude

To test our hypotheses about the additional factors that might explain variation in filling of the potential thermal niche (see Extended Data table 1), we gathered traits for each species in our dataset. Traits were collated from existing databases (FishBase, SeaLifeBase, World Register of Marine Species, IUCN Red List of Threatened Species, AmphibiaWeb, Animal Diversity Web, Meiri 2018, ref [20]) or primary literature, except for three traits: latitudinal midpoint of the realized range, area of the geographic distribution (hereafter range size), and realm. Latitudinal midpoint was calculated as the average of the highest and lowest latitude included in a species' global realized range polygon. Range size was calculated as the number of 1° × 1° grid cells that a species occupied. Species realms were inherited from the thermal tolerance dataset (Supplementary Material of Sunday et al., 2019, ref [2]), but were checked for errors by visually comparing the location of each species' realized range to the assigned realm category.

To test whether body size accounted for variation in potential thermal niche filling, we defined body size as the mean or mean maximum adult length of the species, in cm. If both were available, mean adult length was prioritised for species with determinant growth while mean maximum adult length was prioritised for species with indeterminate growth (e.g., most aquatic species). Body size measurements from existing databases were prioritised, when possible (e.g., FishBase, Meiri 2018, AmphibiaWeb), and otherwise were extracted from the primary literature.

To test whether species that are more dispersal limited fill less of their potential thermal niche, we collected information on each species' dispersal ability. Dispersal distance was defined as how far an individual of a species could be expected to disperse within a generation, assuming that habitat is favourable. We used dispersal distance categories of 0-1 km, 1-10 km, 10+ km, or 100 km+ based on information about migratory distance, studies on dispersal, or home range size. When no such data were available, we used dispersal mode to infer the dispersal distance category of the species. Species were placed in dispersal mode categories based on knowledge of their life histories: walking, crawling, slithering, swimming, non-pelagic development and sessile adults, non-pelagic development and crawling adults, non-pelagic development and swimming adults, pelagic development and sessile adults, pelagic development and crawling adults, pelagic development and swimming adults, pelagic development, and unknown adults. For marine and intertidal species with pelagic development, dispersal distance category was assigned based on information about pelagic larval duration in combination with functional type, informed by Shanks (2009)[21] (see table S7). Dispersal distance category was transformed to a continuous variable for use in models by assigning species the maximum dispersal distance of their category.

We additionally gathered data about each species' seasonal dormancy patterns to better estimate the body temperatures that each species experiences. Cold season dormancy was defined as the ability to be present but avoid the harshest cold temperatures through physiological or behavioural mechanisms (ex. hibernation, burrowing, diapause). Similarly, warm season dormancy was defined as the ability to be present but avoid the harshest warm temperatures through physiological or behavioural mechanisms (ex. aestivation, burrowing). When information on dormancy was unavailable, taxonomic experts inferred this trait or, if this was not possible, we assumed the species was never dormant (see further information in

Supplementary Discussion: *1. Accounting for seasonal dormancy*).

To sensibly bound the potential thermal niche by species' elevation and depth limits, we collected elevational ranges and depth distributions from existing databases (e.g., IUCN Redlist, FishBase) when possible. If such information was unavailable, we inferred it from the highest and lowest reported sampling elevations or depths found in the literature. For marine species still missing depth distribution data, we roughly restricted each species' depth distribution using literature-informed categorical classifications. While coastal, benthic-associated species tend to be limited by depth, pelagic, oceanic species do not. We classified marine species as coastal if from descriptions they were found to be associated with the continental shelf (or depths of < 200m depth), and as benthic if they were found to be substrate-associated. When specific depth distributions were not available for coastal, benthic marine fauna, we restricted their potential thermal niches to areas of the continental shelf (depths of < 200m depth).

Section 5. Plasticity of thermal limits

Many species have phenotypically plastic thermal tolerance limits that allow them to acclimatize to the recently-experienced thermal environment. To account for the effects of acclimatization to local environments, we used data on the thermal acclimation responses of a subset of species in our analysis to examine how acclimatization affects potential thermal niche filling.

To estimate the ability of each species to acclimatize to local temperatures, we compiled the acclimation response ratios (ARRs), or the slope of a linear regression fit to upper or lower thermal limits as a function of experimental acclimation temperature, of ectotherms from published[22–25] papers and databases. We assembled this dataset by searching the literature using Web of Science and Google Scholar for papers published between 2015 and 2020, following the methods used in Bennett et al. [26], which reported studies of organismal thermal limits which included lab acclimation methods. We restricted our dataset to include only studies which included at least two distinct acclimation temperatures so that an ARR could be calculated. Because we used multiple existing compilations of existing data, we checked for duplicates and ARRs of less than -0.15 or greater than 2 were removed because they indicate physiologically harmful acclimation conditions[24]. Multiple ARR estimates for a single species were averaged, leaving a single mean ARR estimate for each species in the dataset.

For species in our analysis that did not have a species-specific ARR available in the dataset, we obtained a coarse estimate of their ARR by calculating a taxonomic Class- or realm-specific ARRs. For species in our dataset assigned to a taxonomic Class that had at least two ARR estimates (n = 230), we calculated a Class-level ARR by aggregating ARR estimates by taxonomic Class for upper (ARR_{upper}) and lower (ARR_{lower}) thermal limits and calculating the mean. For species in our analysis assigned to taxonomic Classes that were unrepresented in the ARR data (n = 18), ARR data were aggregated by realm (*terrestrial, marine*) and the mean realm-level ARR was used in analysis (fig. S6a).

To calculate a species-specific acclimation response intercept for each species in our analysis with thermal tolerance limit assays that reported acclimation temperatures before testing (n=247), we fitted a line with a slope equal to the species' Class- or realm-specific ARR_{upper} or ARR_{lower} through the point representing the species' original fundamental thermal limit and associated acclimation temperature. We then calculated the y-intercept of this line (fig. S6b).

To calculate what the fundamental thermal niche limits of each species would be if it was acclimatized to local conditions in each grid cell, we had to make assumptions about temperatures that species' thermal limits acclimatize to. We assumed that species upper limits acclimatize to the maximum temperature occurring within 7 days before the hottest day ($T_{acc.\ upper}$) and their lower limits acclimatize to the minimum temperature occurring within 7 weeks before the coldest day ($T_{acc.\ lower}$; based on information available on the time course of acclimatization of upper and lower limits[27–29]). We then estimated 'acclimatized' fundamental niche limits in each grid cell using the Class- or realm-level ARRs and the species-specific intercepts as follows:

upper acclimatized fundamental thermal limit = ARR_{upper} x $T_{acc.\ upper}$ + species-specific intercept
lower acclimatized fundamental thermal limit = ARR_{lower} x $T_{acc.\ lower}$ + species-specific intercept

Although this analysis assumes that acclimation capacity does not vary within species, local adaptation in thermal tolerance plasticity across a species range could not explain the thermal niche underfilling patterns observed in this study. While intraspecific variation in acclimation capacity might account for thermal niche underprediction, it cannot theoretically account for thermal niche underfilling. In more variable, higher latitude environments, local adaptation is hypothesized to broaden populations' fundamental thermal niches, which could broaden their potential thermal niches. This might lead to decreased cool niche underprediction and increased warm niche underfilling in higher latitude species, thus strengthening the main pattern we found whereby warm niche underfilling is greater in higher latitude species.

Section 6. Behavioural thermoregulation on land

To ensure we did not overestimate how closely species fill their thermal niche, we assumed in our main analysis that terrestrial species' body temperatures would be equilibrated to the shade when experiencing hot extreme temperatures and to the sun when experiencing cold extreme temperatures. This involved defining the species' extreme body temperatures using the hottest hourly shaded operative temperature and the coldest hourly exposed operative temperature (i.e., operative temperature in the sun). However, we realized these assumptions might lead to overestimates of niche underfilling at the warm or cold edge of the niche if species behaviourally thermoregulate differently than we assumed (i.e., if species prefer to remain in the sun during the hottest hour, or if species prefer to remain in shaded habitat during the coldest hour).

After finding that most species underfilled their warm thermal niche, we wanted to ensure the underfilling we found was not overestimated because of our assumptions about behaviour. (since we found that most species still exhibited cold niche underprediction despite our assumption that they use the warmest habitat available, we did not test for the effects of behaviour on cold niche filling). To test for the effects of our assumptions about behaviour on warm thermal niche filling, we defined species' preferred body temperatures as the body temperatures species maintain in nature[30]. We collected preferred body temperature estimates for terrestrial species from the literature from studies of laboratory assays of temperatures selected in a thermal gradient devoid of ecological constraints (T_{pref}, n = 194), or of field body temperature (T_b, n = 79). For terrestrial species for which we could estimate a preferred body temperature, we adjusted realized upper thermal niche limits to reflect the hottest temperature species would experience in their realized range if allowed to behaviourally thermoregulate towards their

weighted mean preferred temperature by moving between the sun and shade. Three scenarios were possible:

1. *The hottest operative temperature in the shade throughout a species' realized range (original realized niche limit) was hotter than its preferred temperature*. In this case, we set the realized warm niche limit to the hottest operative temperature in the shade.

2. *The hottest operative temperature in the shade throughout a species' realized range was cooler than its preferred temperature.* In this case, we set the species' warm niche limit to its preferred temperature.

3. *The operative temperature in the sun throughout a species' realized range was cooler than its mean preferred temperature.* In this case, we set the species' warm niche limit to the hottest operative temperature in full sun.

Simulating behavioural thermoregulation decreased warm niche underfilling in high-latitude species, indicating that species at high latitudes likely remain in the sun even during the hottest extreme temperatures, however the increase in warm niche filling with latitude remained (Extended Data fig. 6).

Section 7. Accounting for seasonal dormancy

We found that without any correction for seasonal dormancy, realized ranges for many species – mostly at high latitudes – extended to much colder places than potential thermal ranges (i.e., cold tolerance limits underpredict the realized range for many high-latitude species). Since many high-latitude species undergo dormancy during winter, we reasoned that much of this apparent underprediction was likely due to cold-season dormancy. To infer more realistic estimates of species' thermal niches, we decided to use information on species' seasonal dormancy patterns from the literature by masking temperatures during seasons when they are dormant. To ensure we did not bias estimates of thermal niche filling, we corrected for both cold and hot- seasonal dormancy.

We used Yes/No information about species' hot and cold seasonal dormancy patterns collated from the literature to correct the equilibrium body temperatures of species that undergo seasonal dormancy (145 species cold-season dormant, 10 species hot-season dormant). When seasonal dormancy information for species was not available, we assumed no dormancy. We had poor coverage about the duration and timing of dormancy for most species, and therefore took a coarse approach to estimating when species go dormant. We assumed that species known to be seasonally dormant are dormant for the coldest (or hottest) consecutive 6 months of the year (an approximation of dormancy based on a central tendency of around 6 months noted during our data collection). When the dormancy correction was applied, we found cool underprediction was much reduced and so carried out our analyses with more realistic dormancy-corrected potential and realized thermal niches.

To ensure our results were not sensitive to correcting for dormancy, we repeated all analyses on the subset of species in our data that do not undergo dormancy (i.e., no temperatures in their realized or potential thermal niche were masked). We found that analysing only species whose ranges were not corrected for seasonal dormancy (cool niche filling: n=80; warm niche

filling: n=207; range filling: n=61) yielded comparable results to analysing the full dataset of species with and without dormancy (Extended Data fig. 7). While the magnitude of increase in warm niche underfilling with latitude was weakened in terrestrial species when species whose ranges were corrected for dormancy were excluded, this is likely since many species that are dormant during cold seasons live at high latitudes (sparsity of points at high latitudes in Extended Data fig. 7b-c.)

Supplementary Discussion

<u>Section 1. Relationship between range filling and realized range size</u>
We expected that species with larger realized ranges would fill greater proportions of their potential thermal niches and ranges under the hypothesis that larger-ranged species are less ecologically-specialised and thus more directly temperature-limited. We intended to include species' realized range size as a predictor variable in our linear mixed effect model explaining variation in the proportion of a species' range that it fills. However, in the end we excluded range size from the model since we realized the relationship might be affected by our methodology. We restricted potential thermal ranges by continental margins, which could lead to a circular positive relationship between range filling and range size in two ways.

First, in species with large potential ranges that are bounded by continental margins, a shared constraint on potential thermal ranges could lead to greater range filling in larger-ranged species. As range size increases, the amount of area within the continental bound that is left for a species to underfill decreases, which increases the likelihood that the species' will occupy a given area of its potential thermal range[31,32]. This geometric constraint would only influence the relationship between range filling and range size if potential thermal ranges abutted the continental boundaries that they were constrained by. Visual inspection of our potential thermal ranges and their latitudinal extents (see Extended Data fig. 2) indicated that while our potential ranges often do not abut continental margins at the poleward range edge, they often do at the equatorward range edge, meaning that the positive relationship we found between range filling and range size is likely somewhat influenced by this geometric constraint. We note that this pattern whereby the warm, equatorward potential range edge is constrained not by species' thermal tolerance but instead by geometric constraints in species of all range sizes suggests that species share similarly high heat limits, which might constitute an evolutionary attractor (e.g., see ref. [33])

Second, geometric constraints on the potential thermal niche can also impose a lower limit on range filling that would cause a weak positive relationship between range size and range filling. If potential thermal ranges abut continental margins, the area left for the species to underfill would depend on species' range size; larger-ranged species would necessarily fill more of their potential thermal range, and a lower-limit on range filling would be imposed. For example, if you consider two species, one with a small range and another with a broad range. If both species' have potential thermal ranges that are bounded by a continental margin, then the larger-ranged species would necessarily fill more of its potential thermal range. Again, since our data show that potential thermal ranges were somewhat restricted by continental margins, we excluded range size as a covariate in our range filling model as we cannot be sure the positive relationship we found was not influenced by this constraint. In future work, an appropriate null model could be formulated to tease apart the effects of geometric constraints versus ecology on the range filling-range size relationship.

<u>Section 2. Drivers of niche underfilling and climate sensitivity</u>
If biotic limitation or other limiting abiotic niche requirements are responsible for warm niche underfilling, then species might have extra heat tolerance to buffer climate warming at their warm range edge, meaning their warm range edges will not respond directly to temperature change. However, if warm niche underfilling occurs because laboratory-tested individual heat

limits do not represent the limits of population growth, then species could have high heat sensitivity at their warm range edge despite findings of apparent thermal safety.

References

1. Soberón, J. & Arroyo-Peña, B. Are fundamental niches larger than the realized? Testing a 50-year-old prediction by Hutchinson. *PLOS ONE* **12**, e0175138 (2017).
2. Sunday, J. *et al.* Thermal tolerance patterns across latitude and elevation. *Philos. Trans. R. Soc. B Biol. Sci.* **374**, 20190036 (2019).
3. GBIF. The Global Biodiversity Information Facility. (2020).
4. Cogger, H. *Reptiles and Amphibians of Australia*. (CSIRO PUBLISHING, 2018).
5. Spalding, M. D. *et al.* Marine Ecoregions of the World: A Bioregionalization of Coastal and Shelf Areas. *BioScience* **57**, 573–583 (2007).
6. Olson, D. M. *et al.* Terrestrial Ecoregions of the World: A New Map of Life on Earth. *BioScience* **51**, 933 (2001).
7. Camacho, A., Trefaut Rodrigues, M. & Navas, C. Extreme operative temperatures are better descriptors of the thermal environment than mean temperatures. *J. Therm. Biol.* **49–50**, 106–111 (2015).
8. Sears, M. W. Geographic variation in the life history of the sagebrush lizard: the role of thermal constraints on activity. *Oecologia* **143**, 25–36 (2005).
9. Sinervo, B. *et al.* Erosion of Lizard Diversity by Climate Change and Altered Thermal Niches. *Science* **328**, 894–899 (2010).
10. Kearney, M. R. & Porter, W. P. NicheMapR – an R package for biophysical modelling: the microclimate model. *Ecography* **40**, 664–674 (2017).
11. Campbell, G. S. & Norman, J. M. Animals and their Environment. in *An Introduction to Environmental Biophysics* 185–207 (Springer New York, 1998). doi:10.1007/978-1-4612-1626-1_12.
12. Buckley, L. B. Linking Traits to Energetics and Population Dynamics to Predict Lizard Ranges in Changing Environments. *Am. Nat.* **171**, E1–E19 (2008).
13. Spotila, J. R. & Berman, E. N. Determination of skin resistance and the role of the skin in controlling water loss in amphibians and reptiles. *Comp. Biochem. Physiol. A Physiol.* **55**, 407–411 (1976).
14. Mautz, W. J. Factors influencing evaporative water loss in lizards. *Comp. Biochem. Physiol. A Physiol.* **67**, 429–437 (1980).
15. Kearney, M., Shine, R. & Porter, W. P. The potential for behavioral thermoregulation to buffer "cold-blooded" animals against climate warming. *Proc. Natl. Acad. Sci.* **106**, 3835–3840 (2009).
16. Algar, A. C., Morley, K. & Boyd, D. S. Remote sensing restores predictability of ectotherm body temperature in the world's forests: XXXX. *Glob. Ecol. Biogeogr.* **27**, 1412–1425 (2018).
17. Hertz, P. E., Huey, R. B. & Stevenson, R. D. Evaluating Temperature Regulation by Field-Active Ectotherms: The Fallacy of the Inappropriate Question. *Am. Nat.* **142**, 796–818 (1993).
18. Kearney, M. R. & Enriquez-Urzelai, U. A general framework for jointly modelling thermal and hydric constraints on developing eggs. *Methods Ecol. Evol.* 2041–210X.14018 (2022) doi:10.1111/2041-210X.14018.
19. Gates, D. M. *Biophysical Ecology*. (Springer New York, 1980). doi:10.1007/978-1-4612-6024-0.
20. Meiri, S. Traits of lizards of the world: Variation around a successful evolutionary design. *Glob. Ecol. Biogeogr.* **27**, 1168–1172 (2018).

21. Shanks, A. L. Pelagic Larval Duration and Dispersal Distance Revisited. *Biol. Bull.* **216**, 373–385 (2009).
22. Rohr, J. R. *et al.* The complex drivers of thermal acclimation and breadth in ectotherms. *Ecol. Lett.* **21**, 1425–1439 (2018).
23. Comte, L. & Olden, J. D. Climatic vulnerability of the world's freshwater and marine fishes. *Nat. Clim. Change* **7**, 718–722 (2017).
24. Gunderson, A. R. & Stillman, J. H. Plasticity in thermal tolerance has limited potential to buffer ectotherms from global warming. *Proc. R. Soc. B Biol. Sci.* **282**, 20150401 (2015).
25. Morley, S. A., Peck, L. S., Sunday, J. M., Heiser, S. & Bates, A. E. Physiological acclimation and persistence of ectothermic species under extreme heat events. *Glob. Ecol. Biogeogr.* **28**, 1018–1037 (2019).
26. Bennett, J. M. *et al.* GlobTherm, a global database on thermal tolerances for aquatic and terrestrial organisms. *Sci. Data* **5**, (2018).
27. Gunderson, A. R., Dillon, M. E. & Stillman, J. H. Estimating the benefits of plasticity in ectotherm heat tolerance under natural thermal variability. *Funct. Ecol.* **31**, 1529–1539 (2017).
28. Pintor, A. F. V., Schwarzkopf, L. & Krockenberger, A. K. Extensive Acclimation in Ectotherms Conceals Interspecific Variation in Thermal Tolerance Limits. *PLOS ONE* **11**, e0150408 (2016).
29. Weldon, C. W., Terblanche, J. S. & Chown, S. L. Time-course for attainment and reversal of acclimation to constant temperature in two Ceratitis species. *J. Therm. Biol.* **36**, 479–485 (2011).
30. Light, P., Dawson, W. R., Shoemaker, V. H. & Main, A. R. Observations on the Thermal Relations of Western Australian Lizards. *Copeia* **1966**, 97 (1966).
31. Estrada, A., Morales-Castilla, I., Meireles, C., Caplat, P. & Early, R. Equipped to cope with climate change: traits associated with range filling across European taxa. *Ecography* **41**, 770–781 (2018).
32. Seliger, B. J., McGill, B. J., Svenning, J. & Gill, J. L. Widespread underfilling of the potential ranges of North American trees. *J. Biogeogr.* **48**, 359–371 (2021).
33. Bennett, J. M. *et al.* The evolution of critical thermal limits of life on Earth. *Nat. Commun.* **12**, 1198 (2021).

Supplementary Figures

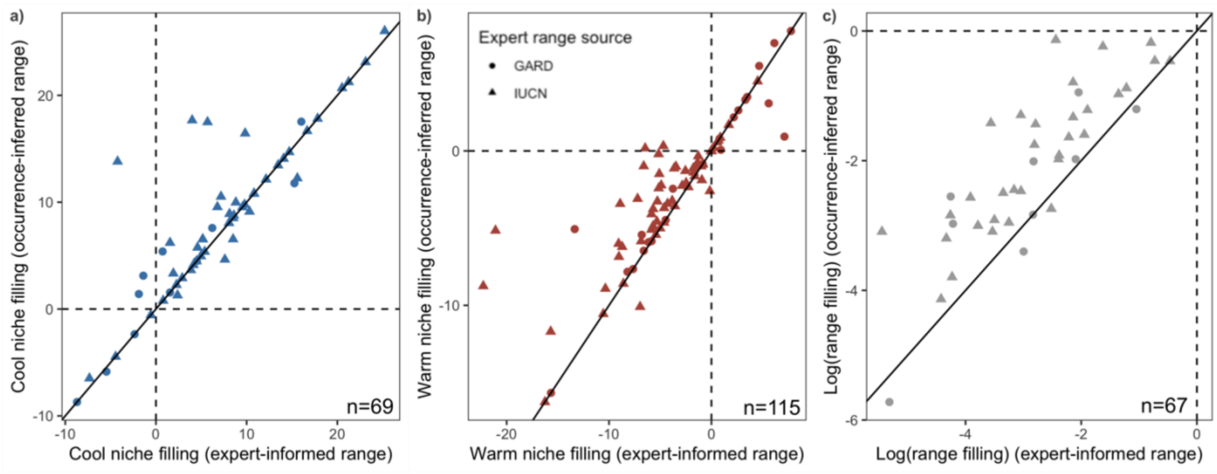

Fig. S1. Comparison of potential thermal niche filling values when realized ranges from different sources are used. a-c. To ensure measurements of potential thermal niche filling were not sensitive to whether we used an expert-informed realized range polygon (from the IUCN or GARD databases) or a polygon inferred from GBIF occurrence data, we inspected the variation in potential thermal niche filling values caused by the source of the realized range. For species for which we had both an expert IUCN and inferred GBIF range map (n = 85; triangles) or both an expert GARD and inferred GBIF range map (n = 34; circles), we compared thermal niche filling values measured using the expert-informed range to values measured using the range inferred from GBIF occurrence data. Little deviation from the 1:1 line (solid, diagonal line) and no obvious bias indicates that thermal niche and range filling values were similar, regardless of the source of realized range.

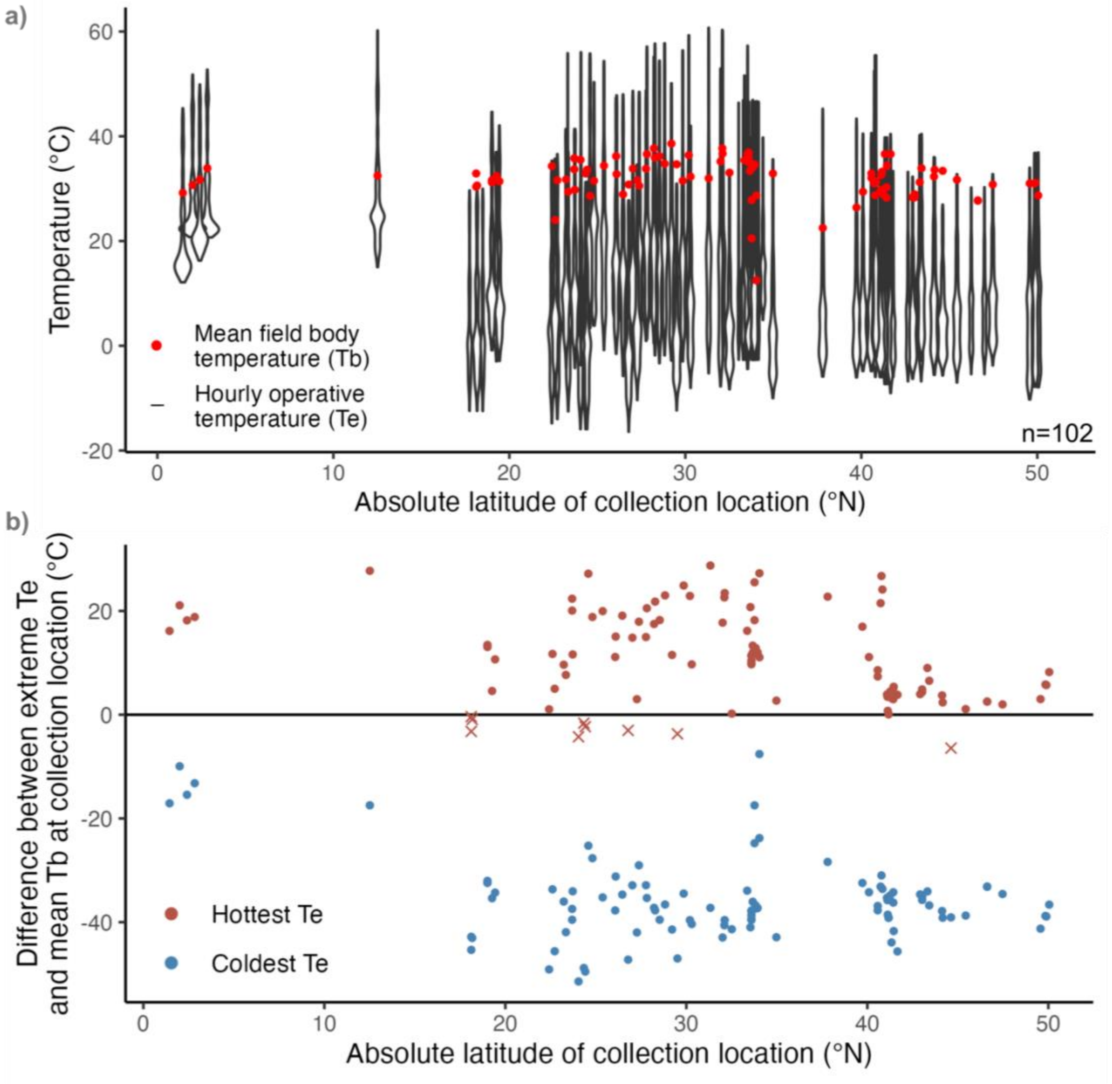

Fig. S2. Mean field body temperature of lizards fall within the range of operative body temperatures estimated different collection locations across latitude. We used a published dataset of lizard mean field body temperatures (T_b, Algar et al. 2018[16]) to validate our estimates of terrestrial species' operative body temperatures (T_e),. **a.** For each available T_b estimate for a population with a known collection location of a species that appears in our data (n=102), we compared the reported T_b (red dots) to the distribution of modelled hourly T_e at the collection location (black distributions). For most populations sampled (n=93), T_b was within the range of T_e estimates. **b.** We looked at the difference between extreme T_e and T_b across latitude, we found that the difference between T_b and the coldest T_e increased with latitude (Fig S10b), likely because lizards spend more time in the sun at higher latitudes. We found no latitudinal pattern in the cases where T_b was hotter than the hottest T_e at the collection location (cross symbols).

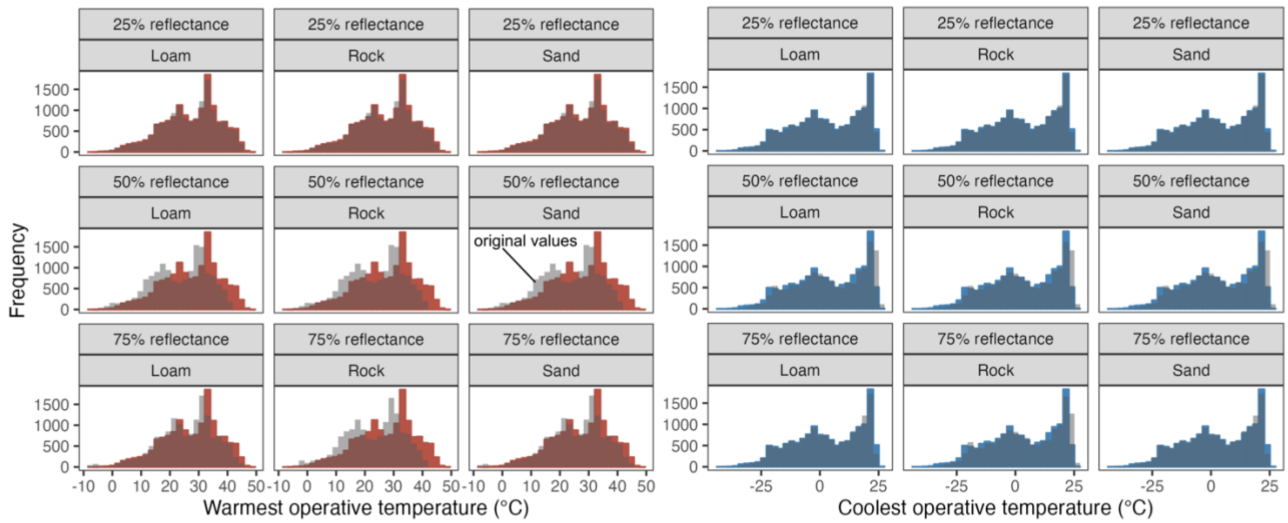

Fig. S3. Distribution of warmest and coolest operative temperatures for *Pardosa nigriceps* when model parameters are allowed to vary. We tested the sensitivity of our results to variation in model parameters by comparing the warmest and coldest operative temperatures that five species in our dataset would experience in every grid cell if soil reflectance, soil type, burrow depth, and skin absorbance and reflectance are held constant (grey distributions) to those they would experience if these parameters were allowed to vary (coloured distributions, see *Supplementary Methods*). Results for only one species (*Pardosa nigriceps*) are shown. While allowing parameters to vary hardly changed the distributions of cool operative temperatures, distributions of warm operative temperatures shifted slightly towards hotter temperatures.

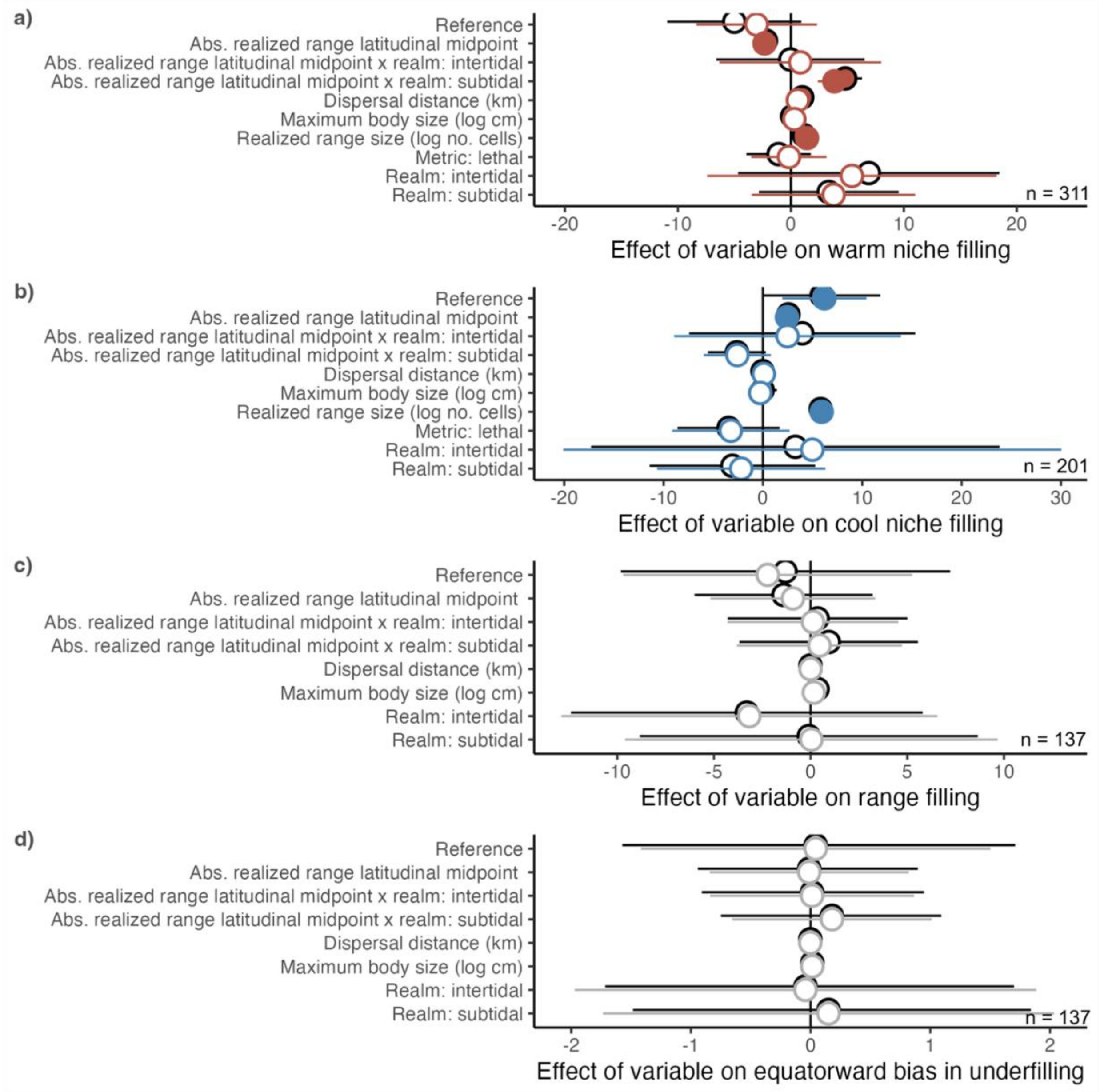

Fig. S4. Parameter estimates from linear mixed effect models do not substantially differ from those from phylogenetic least squares models. a-d. Results of analyses on the subset of species for which a time-calibrated phylogeny could be estimated (n = 376 species) are not sensitive to the method used to account for non-independence of data due to shared evolutionary history. Comparing parameter estimates (dots) from linear mixed effect models including nested taxonomic ranks as a random effect (coloured) to those estimated using phylogenetic generalized least squares models (black) for models of warm niche filling (panel a; n = 311 species), cool niche filling (panel b; n = 201 species), potential range filling (panel c; n = 137 species), and bias in range underfilling (panel d; n = 137 species) show that the linear mixed effect modelling framework sufficiently captures variation that is due to shared evolutionary history. Solid dots denote parameter estimates whose 95% confidence (whiskers) intervals do not overlap zero. The reference level for realm is terrestrial.

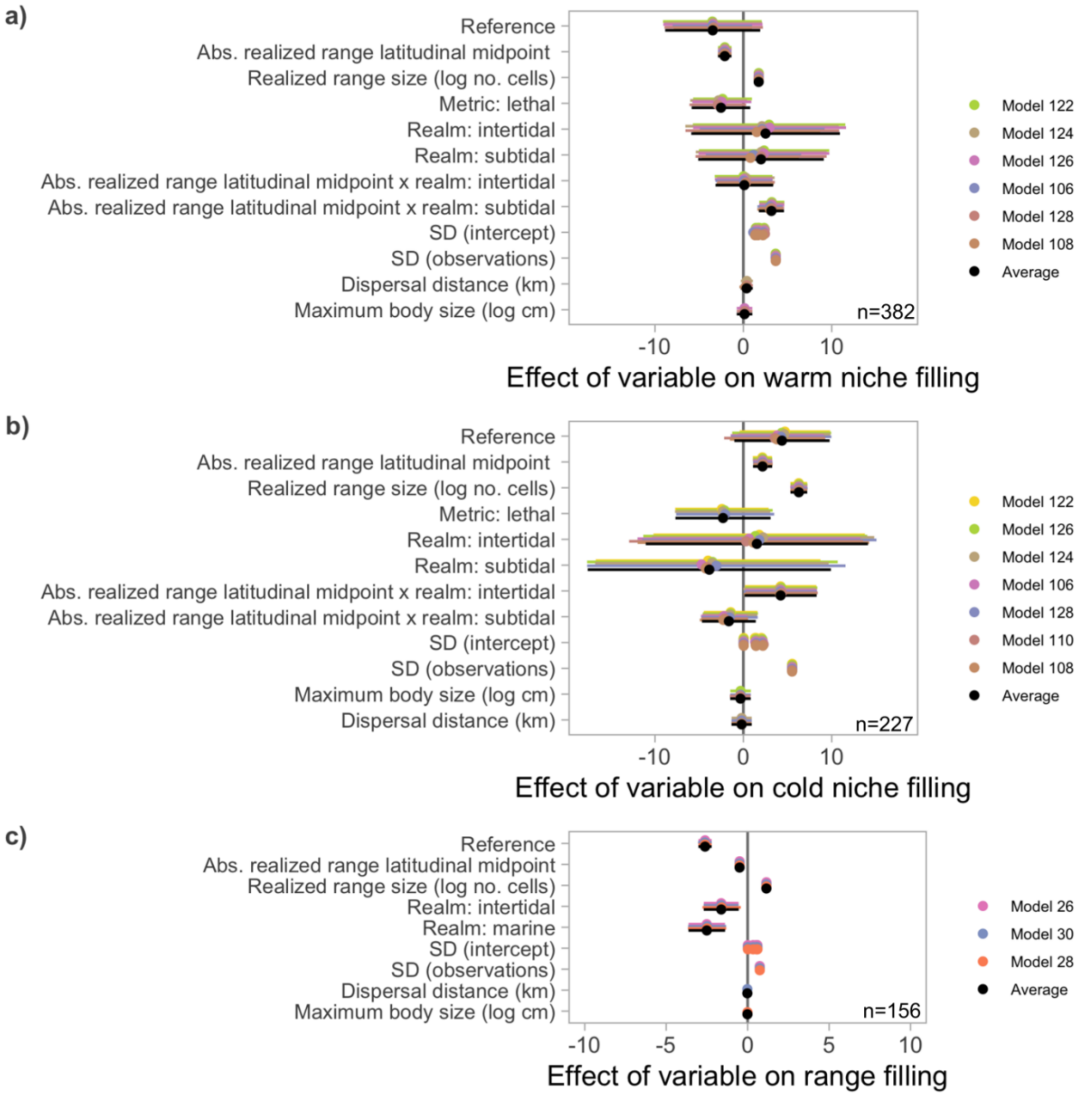

Fig. S5. Dot and whisker plot of parameter estimates of models in 95% confidence set compared to overall model average. a-c. Comparing the parameter estimates of individual models included in the 95 % confidence set (coloured) to the model-averaged parameters (black) for linear mixed effects models of warm niche filling (panel a; n = 382 species), cool niche filling (panel b; n = 227 species), and potential range filling (panel c; n = 156 species) shows that the exclusion of certain explanatory variables from some models in the top set does not dramatically affect the estimation of other model parameters. Results from the model fit to equatorward bias in underfilling are not shown here because a single model comprised the 95% confidence set.

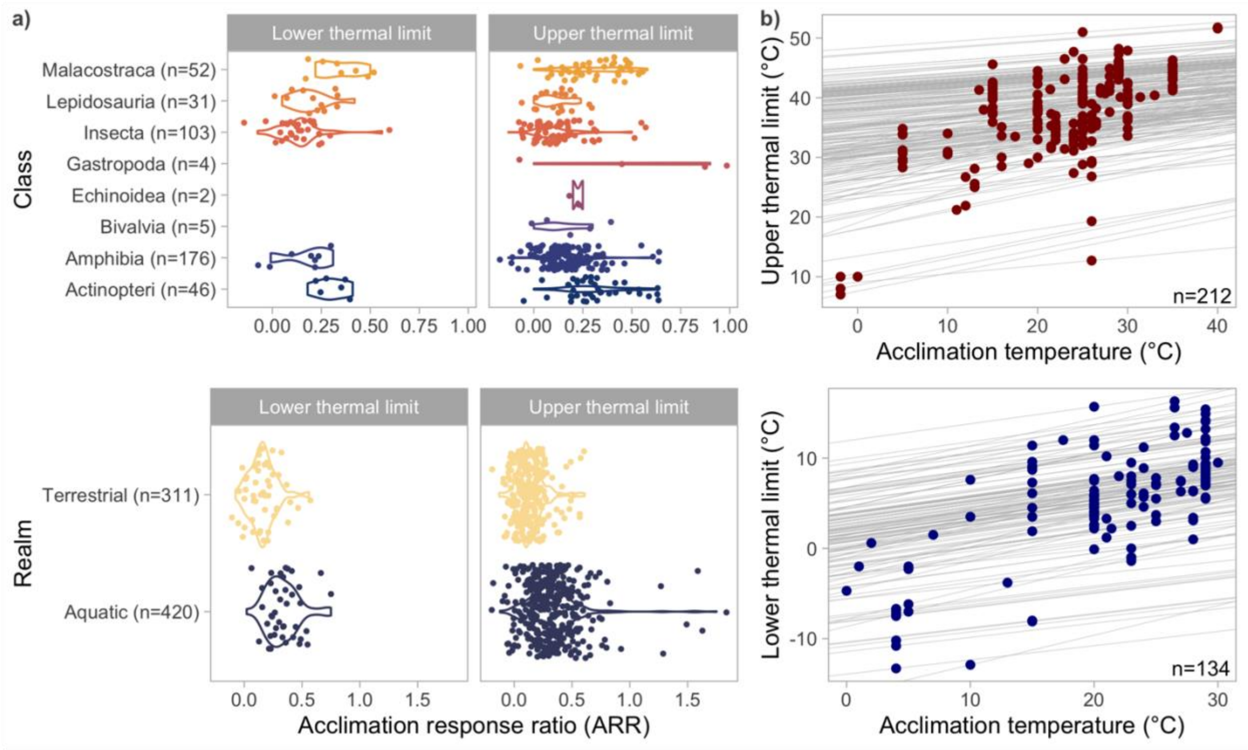

Fig. S6. Class- and realm-specific acclimation response ratios used to estimate fundamental thermal niche limits of acclimatized species. a. We collated acclimation response ratio (ARR) estimates for upper and low thermal tolerance limits from pre-exi[1–4]sting databases and used them to calculate an average ARR at the taxonomic Class- and realm level. b. For species in our data for which the acclimation temperature before thermal limit testing was known, we fit a line with a slope defined by the species Class- or realm-specific through the species original thermal tolerance limit (points) and used the equation of this line to calculate the acclimatized species' fundamental thermal limits according to environmental temperatures.

Supplementary Tables

Table S1. Model fitted relationships between thermal tolerance breadth and latitude and warm niche underfilling and thermal tolerance breadth across realms. Parameters of linear models describing how thermal tolerance breadth changes with latitude (a) and how warm thermal niche filling changes with thermal tolerance breadth (b) across realms. Models were fit to only the subset of species with both thermal tolerance limits (n = 185). A single asterisk (*) denotes a *p*-value less than 0.05, and double (**) signals a *p*-value less than 0.01.

**a. Thermal tolerance breadth ~ latitude*realm**					
**fixed effect**	**effect type**	**estimate**	**s.e.**	***t*-value**	***p*-value**
reference	intercept	25.65	1.02	25.04	<0.001**
abs. realized range latitudinal midpoint	slope	0.31	0.03	10.29	<0.001**
abs. realized range latitudinal midpoint x realm: intertidal	slope	-0.21	0.08	-2.58	0.01*
abs. realized range latitudinal midpoint x realm: subtidal	slope	-0.19	0.08	-2.36	0.02*
realm: intertidal	intercept	6.50	3.73	1.74	0.08
realm: subtidal	intercept	-2.17	2.48	-0.88	0.38
**b. Warm niche filling ~ thermal tolerance breadth*realm**					
fixed effect	effect type	estimate	s.e.	*t*-value	*p*-value
reference	intercept	15.94	2.33	6.85	<0.001**
thermal tolerance breadth	slope	-0.56	0.06	-8.72	<0.001**
thermal tolerance breadth x realm: intertidal	slope	0.30	0.16	1.88	0.06
thermal tolerance breadth x realm: subtidal	slope	0.50	0.31	1.62	0.11
realm: intertidal	intercept	-5.77	5.85	-0.99	0.32
realm: subtidal	intercept	-15.43	8.46	-1.82	0.07

Table S2. Model results comparing warm and cool potential thermal niche filling with and without acclimatization. Model-averaged parameters for warm and cool potential thermal niche filling when acclimatization is (a) and is not (b) allowed for a subset of species with both thermal limits for which acclimation ability could be estimated. A single asterisk (*) denotes a *p*-value less than 0.05, and double (**) signals a *p*-value less than 0.01.

			a) variation in acclimation ability				b) no variation in acclimation ability			
	fixed effect	effect type	estimate	s.e.	z-value	p-value	estimate	s.e.	z-value	p-value
warm niche edge n=212	reference	intercept	-6.98	2.31	2.98	0.003**	-4.06	1.38	2.89	0.004*
	abs. realized range latitudinal midpoint	slope	-2.52	0.42	5.9	<0.001**	-3.1	0.44	6.97	<0.001**
	abs. realized range latitudinal midpoint x realm: intertidal	slope	2.33	1.44	1.59	0.111	4.34	1.42	3.01	0.003**
	abs. realized range latitudinal midpoint x realm: subtidal	slope	3.2	0.64	4.94	<0.001**	3.81	0.68	5.56	<0.001**
	realized range size (log no. cells)	slope	1.18	0.3	3.8	<0.001**	0.88	0.37	2.35	0.019*
	dispersal distance (km)	slope	-0.01	0.14	0.08	0.934	0.01	0.15	0.04	0.97
	maximum body size (log cm)	slope	-0.04	0.25	0.15	0.879	0.16	0.34	0.46	0.649
	realm: intertidal	intercept	2.89	3.35	0.85	0.396	2.25	2.53	0.88	0.381
	realm: subtidal	intercept	5.08	2.95	1.7	0.09	2.24	1.96	1.13	0.26
	metric: lethal	intercept	0.2	3.6	0.06	0.956	2.21	1.53	1.41	0.158
cool niche edge n=134	reference	intercept	0.2	3.6	0.06	0.956	2.15	3.69	0.57	0.568
	abs. realized range latitudinal midpoint	slope	3.86	0.76	4.96	<0.001**	3.9	0.73	5.26	<0.001**
	abs. realized range latitudinal midpoint x realm: intertidal)	slope	3.49	2.43	1.37	0.172	2.74	2.27	1.15	0.249
	abs. realized range latitudinal midpoint x realm: subtidal)	slope	-3.67	1.68	2.1	0.036*	-4.94	1.64	2.88	0.004**
	realized range size (log no. cells)	slope	6.35	0.6	10.38	<0.001**	6.41	0.57	10.93	<0.001**
	dispersal distance (km)	slope	-0.4	0.67	0.59	0.553	6.41	0.57	0.47	0.64
	maximum body size (log cm)	slope	-0.59	0.85	0.69	0.491	-0.63	0.84	0.75	0.456
	realm: intertidal	intercept	4.59	0.85	0.69	0.491	5.02	6.59	0.58	0.56
	realm: subtidal	intercept	0.8	6.59	0.54	0.592	0.8	7.41	0.08	0.934
	metric: lethal	intercept	-1.25	2.59	0.46	0.644	-0.46	2.47	0.18	0.859

Table S3. Model results comparing warm thermal niche filling with and without behavioural thermoregulation. Model-averaged parameters for warm potential thermal niche filling when behaviour is (a) and is not (b) allowed for a subset of terrestrial species for which thermal preference data were available in the literature (n = 219). A single asterisk (*) denotes a *p*-value less than 0.05, and double (**) signals a *p*-value less than 0.01.

			a) behavioural thermoregulation				b) no behavioural thermoregulation			
	**fixed effect**	**effect type**	**estimate**	**s.e.**	**z-value**	**p-value**	**estimat**	**s.e.**	**z-value**	**p-value**
warm niche edge n=219	reference	intercept	-1.67	0.61	2.73	0.006**	-2.46	0.75	3.25	0.001**
	abs. realized range latitudinal midpoint	slope	-1.1	0.25	4.32	<0.001**	-1.95	0.35	5.49	<0.001**
	realized range size (log no. cells)	slope	1.09	0.24	4.53	<0.001**	1.88	0.33	5.55	<0.001**
	dispersal distance (km)	slope	0.11	0.19	0.57	0.57	0.07	0.2	0.38	0.703
	maximum body size (log cm)	slope	0.27	0.36	0.75	0.453	0.26	0.41	0.64	0.522
	metric: lethal	intercept	0.23	2.18	0.1	0.918	0.35	3.36	0.1	0.917

Table S4. Model results comparing range filling with and without acclimatization. Model-averaged parameters for range filling when acclimatization is (a) and is not (b) allowed for a subset of species with both thermal limits for which acclimation ability could be estimated (n = 90). A single asterisk (*) denotes a *p*-value less than 0.05, and double (**) signals a *p*-value less than 0.01.

a) Range filling									
		a) variation in acclimation ability				***b) no variation in acclimation ability***			
fixed effects	**effect type**	**estimate**	**s.e.**	**z-value**	**p-value**	**estimate**	**s.e.**	**z-value**	**p-value**
reference	intercept	-2.62	0.47	5.35	<0.001	-2.63	0.5	5.11	<0.001 *
abs. realized range latitudinal midpoint	slope	0.05	0.14	0.35	0.723	-0.05	0.15	0.34	0.735
abs. realized range latitudinal midpoint realm: intertidal	slope	-0.05	0.31	0.14	0.887	0	0.25	0	0.997
abs. realized range latitudinal midpoint realm: subtidal	slope	-0.37	0.65	0.56	0.577	-0.17	0.45	0.37	0.712
dispersal distance (km)	slope	0.03	0.12	0.28	0.778	0.02	0.1	0.16	0.87
maximum body size (log cm)	slope	0.01	0.09	0.09	0.925	0.01	0.09	0.08	0.933
realm: intertidal	intercept	-1.83	1.23	0.92	0.355	-1.77	1.28	0.87	0.385
realm: subtidal	intercept	-2.84	0.8	2.21	0.027*	-2.65	0.93	1.91	0.057
b) Equatorward bias in underfilling									
		a) variation in acclimation ability				***b) no variation in acclimation ability***			
fixed effects	**effect type**	**estimate**	**s.e.**	**z-value**	**p-value**	**estimate**	**s.e.**	**z-value**	**p-value**
reference	intercept	0.16	0.06	2.75	0.009**	-0.04	0.17	-0.27	0.792
abs. realized range latitudinal midpoint	slope	0.05	0.04	1.31	0.199	0.01	0	2.06	0.048*
abs. realized range latitudinal midpoint x realm: intertidal	slope	-0.05	0.11	-0.45	0.655	-0.01	0.01	-0.8	0.434
abs. realized range latitudinal midpoint x realm: subtidal	slope	-0.04	0.08	-0.55	0.585	-0.01	0.01	-0.93	0.364
realm: intertidal	intercept	-0.14	0.18	-0.81	0.475	0.07	0.46	0.15	0.889
realm: subtidal	intercept	-0.15	0.14	-1.09	0.355	0.04	0.27	0.14	0.897

Table S5. Criteria used to systematically assign dispersal distance categories to species with pelagic larval development (PLD). Summary of information about pelagic larval duration and functional type from Shanks, 2009 used to assign marine and intertidal species with pelagic development to dispersal distance categories.

Description of species functional group	*Assigned dispersal distance category*
• PLD < 12 h<br>• Clonal species of unknown PLD<br>• Larval brooders with little mobility (ex. sessile)	0-1
• PLD 12 h to 1 week<br>• PLD 1 week to 30 d AND benthic larvae<br>• Benthic larvae of unknown PLD<br>• Coastal, intertidal or reef species with unknown larval behaviour (no evidence that it is planktonic) and unknown PLD<br>• Larval brooders with higher mobility and no other info on dispersal (ex. fish)<br>• Lecithotrophic pelagic larvae and no other info	1-10
• PLD 1 week to 30 d<br>• Open ocean species with PLD 1 week to 30 days or pelagic/planktonic larvae of unknown PLD<br>• Coastal, intertidal or reef species with planktonic larva of and unknown PLD or PLD of 1 week or more<br>• Benthic crustaceans with planktonic larvae	10-100
• Open ocean species (> 30m depth) with PLD > 1 month	100+

Table S6. List of species included in final models. Note that some species in this list (n=10) were included in no models because although we had enough information to analyze their thermal niches, some of their traits were unknown.

	Scientific name	Warm niche filling	Cool niche filling	Range filling	Bias in underfilling
1	*Agkistrodon contortrix*	x	x	x	x
2	*Agroeca proxima*	x	x	x	x
3	*Ameiva festiva*	x	x	x	x
4	*Amphibolurus muricatus*	x	x	x	x
5	*Anodontia bullula*	x	x	x	x
6	*Anolis barbouri*	x	x	x	x
7	*Anolis carolinensis*	x	x	x	x
8	*Anolis cupreus*	x	x	x	x
9	*Anolis humilis*	x	x	x	x
10	*Anolis intermedius*	x	x	x	x
11	*Anolis lemurinus*	x	x	x	x
12	*Anolis limifrons*	x	x	x	x
13	*Anolis lionotus*	x	x	x	x
14	*Anolis tropidolepis*	x	x	x	x
15	*Austrelaps superbus*	x	x	x	x
16	*Barbatia pistachia*	x	x	x	x
17	*Bassiana duperreyi*	x	x	x	x
18	*Cerastoderma edule*	x	x	x	x
19	*Chamaeleo dilepis*	x	x	x	x
20	*Chamaeleo jacksonii*	x	x	x	x
21	*Clubiona diversa*	x	x	x	x
22	*Clubiona trivialis*	x	x	x	x
23	*Coleonyx brevis*	x	x	x	x
24	*Coleonyx variegatus*	x	x	x	x
25	*Cophosaurus texanus*	x	x	x	x
26	*Cordylus cordylus*	x	x	x	x
27	*Cordylus oelofseni*	x	x	x	x
28	*Crustulina guttata*	x	x	x	x
29	*Ctenophorus decresii*	x	x	x	x
30	*Ctenotus taeniolatus*	x	x	x	x
31	*Ctenotus uber*	x	x	x	x
32	*Dascyllus aruanus*	x	x	x	x
33	*Dendrobates auratus*	x	x	x	x
34	*Desmognathus fuscus*	x	x	x	x
35	*Dipsosaurus dorsalis*	x	x	x	x
36	*Egernia cunninghami*	x	x	x	x
37	*Egernia saxatilis*	x	x	x	x
38	*Egernia striolata*	x	x	x	x
39	*Eleutherodactylus coqui*	x	x	x	x
40	*Elgaria multicarinata*	x	x	x	x

41	*Engraulis japonicus*	x	x	x	x
42	*Eremias argus*	x	x	x	x
43	*Eremias brenchleyi*	x	x	x	x
44	*Eremiascincus fasciolatus*	x	x	x	x
45	*Eremiascincus richardsoni*	x	x	x	x
46	*Eulamprus heatwolei*	x	x	x	x
47	*Eulamprus kosciuskoi*	x	x	x	x
48	*Eulamprus quoyii*	x	x	x	x
49	*Eulamprus tympanum*	x	x	x	x
50	*Eurycea bislineata*	x	x	x	x
51	*Fundulus parvipinnis*	x	x	x	x
52	*Gehyra variegata*	x	x	x	x
53	*Halichoeres dispilus*	x	x	x	x
54	*Hemachatus haemachatus*	x	x	x	x
55	*Hemidactylus frenatus*	x	x	x	x
56	*Hemidactylus turcicus*	x	x	x	x
57	*Hemiergis decresiensis*	x	x	x	x
58	*Heteronotia binoei*	x	x	x	x
59	*Lacerta agilis*	x	x	x	x
60	*Lampropholis delicata*	x	x	x	x
61	*Lampropholis guichenoti*	x	x	x	x
62	*Lepidodactylus lugubris*	x	x	x	x
63	*Lepidophyma flavimaculatum*	x	x	x	x
64	*Linepithema humile*	x	x	x	x
65	*Liolaemus albiceps*	x	x	x	x
66	*Liolaemus capillitas*	x	x	x	x
67	*Liolaemus chacoensis*	x	x	x	x
68	*Liolaemus cuyanus*	x	x	x	x
69	*Liolaemus darwinii*	x	x	x	x
70	*Liolaemus dorbignyi*	x	x	x	x
71	*Liolaemus irregularis*	x	x	x	x
72	*Liolaemus koslowskyi*	x	x	x	x
73	*Liolaemus laurenti*	x	x	x	x
74	*Liolaemus melanops*	x	x	x	x
75	*Liolaemus multicolor*	x	x	x	x
76	*Liolaemus olongasta*	x	x	x	x
77	*Liolaemus petrophilus*	x	x	x	x
78	*Liolaemus poecilochromus*	x	x	x	x
79	*Liolaemus pseudoanomalus*	x	x	x	x
80	*Liolaemus quilmes*	x	x	x	x
81	*Liolaemus riojanus*	x	x	x	x
82	*Liolaemus robertmertensi*	x	x	x	x
83	*Liolaemus rothi*	x	x	x	x
84	*Liolaemus salinicola*	x	x	x	x
85	*Liolaemus scapularis*	x	x	x	x
86	*Littorina littorea*	x	x	x	x

87	*Littorina obtusata*	x	x	x	x
88	*Littorina saxatilis*	x	x	x	x
89	*Lophognathus gilberti*	x	x	x	x
90	*Macoma balthica*	x	x	x	x
91	*Malacoctenus zonifer*	x	x	x	x
92	*Menidia menidia*	x	x	x	x
93	*Moloch horridus*	x	x	x	x
94	*Mytilus edulis*	x	x	x	x
95	*Nannoscincus maccoyi*	x	x	x	x
96	*Niveoscincus metallicus*	x	x	x	x
97	*Niveoscincus ocellatus*	x	x	x	x
98	*Notechis scutatus*	x	x	x	x
99	*Nucella lapillus*	x	x	x	x
100	*Oedothorax apicatus*	x	x	x	x
101	*Oncorhynchus tshawytscha*	x	x	x	x
102	*Pardosa nigriceps*	x	x	x	x
103	*Periophthalmus kalolo*	x	x	x	x
104	*Philodromus aureolus*	x	x	x	x
105	*Phrynosoma cornutum*	x	x	x	x
106	*Phymaturus antofagastensis*	x	x	x	x
107	*Plagiotremus azaleus*	x	x	x	x
108	*Platysaurus intermedius*	x	x	x	x
109	*Plestiodon chinensis*	x	x	x	x
110	*Plestiodon elegans*	x	x	x	x
111	*Plestiodon gilberti*	x	x	x	x
112	*Podarcis muralis*	x	x	x	x
113	*Prinerigone vagans*	x	x	x	x
114	*Protobothrops mucrosquamatus*	x	x	x	x
115	*Psammodromus algirus*	x	x	x	x
116	*Psammodromus hispanicus*	x	x	x	x
117	*Pseudacris cadaverina*	x	x	x	x
118	*Pseudacris regilla*	x	x	x	x
119	*Pseudemoia entrecasteauxii*	x	x	x	x
120	*Pseudemoia spenceri*	x	x	x	x
121	*Pseudonaja textilis*	x	x	x	x
122	*Rana cascadae*	x	x	x	x
123	*Rana pretiosa*	x	x	x	x
124	*Rankinia diemensis*	x	x	x	x
125	*Saproscincus mustelina*	x	x	x	x
126	*Sceloporus graciosus*	x	x	x	x
127	*Sceloporus malachiticus*	x	x	x	x
128	*Sceloporus merriami*	x	x	x	x
129	*Sceloporus occidentalis*	x	x	x	x
130	*Sceloporus undulatus*	x	x	x	x
131	*Sceloporus variabilis*	x	x	x	x
132	*Scotina gracilipes*	x	x	x	x

133	*Spea hammondii*	x	x	x	x
134	*Sphenomorphus incognitus*	x	x	x	x
135	*Sphenomorphus indicus*	x	x	x	x
136	*Sphenomorphus taiwanensis*	x	x	x	x
137	*Sphoeroides maculatus*	x	x	x	x
138	*Stemonyphantes lineatus*	x	x	x	x
139	*Takydromus hsuehshanensis*	x	x	x	x
140	*Takydromus septentrionalis*	x	x	x	x
141	*Takydromus sexlineatus*	x	x	x	x
142	*Testudo hermanni*	x	x	x	x
143	*Thalassoma lucasanum*	x	x	x	x
144	*Thamnophis elegans*	x	x	x	x
145	*Thamnophis ordinoides*	x	x	x	x
146	*Thamnophis sirtalis*	x	x	x	x
147	*Tiliqua nigrolutea*	x	x	x	x
148	*Tiliqua rugosa*	x	x	x	x
149	*Tlalocohyla smithii*	x	x	x	x
150	*Trachylepis striata*	x	x	x	x
151	*Trimeresurus gracilis*	x	x	x	x
152	*Trimeresurus stejnegeri*	x	x	x	x
153	*Uta stansburiana*	x	x	x	x
154	*Varanus varius*	x	x	x	x
155	*Xantusia vigilis*	x	x	x	x
156	*Zootoca vivipara*	x	x	x	x
157	*Anolis cristatellus*	x	x		
158	*Cordylus niger*	x	x		
159	*Ctenotus regius*	x	x		
160	*Eremias multiocellata*	x	x		
161	*Hemiergis peroni*	x	x		
162	*Hemigrapsus nudus*	x	x		
163	*Liolaemus abaucan*	x	x		
164	*Liolaemus bibronii*	x	x		
165	*Liolaemus boulengeri*	x	x		
166	*Liolaemus canqueli*	x	x		
167	*Liolaemus elongatus*	x	x		
168	*Liolaemus fitzingerii*	x	x		
169	*Liolaemus kingii*	x	x		
170	*Liolaemus kriegi*	x	x		
171	*Liolaemus multimaculatus*	x	x		
172	*Liolaemus telsen*	x	x		
173	*Liolaemus xanthoviridis*	x	x		
174	*Nacella concinna*	x	x		
175	*Phymaturus dorsimaculatus*	x	x		
176	*Phymaturus indistinctus*	x	x		
177	*Phymaturus palluma*	x	x		
178	*Phymaturus patagonicus*	x	x		

179	*Phymaturus somuncurensis*	x	x
180	*Phymaturus tenebrosus*	x	x
181	*Phymaturus zapalensis*	x	x
182	*Tautogolabrus adspersus*	x	x
183	*Trapelus savignii*	x	x
184	*Acar domingensis*	x	
185	*Acontias meleagris*	x	
186	*Adamussium colbecki*	x	
187	*Adelotus brevis*	x	
188	*Ambystoma jeffersonianum*	x	
189	*Ambystoma macrodactylum*	x	
190	*Ambystoma maculatum*	x	
191	*Anaxyrus compactilis*	x	
192	*Anniella pulchra*	x	
193	*Anolis auratus*	x	
194	*Anolis cybotes*	x	
195	*Anolis distichus*	x	
196	*Anolis frenatus*	x	
197	*Anolis marcanoi*	x	
198	*Anolis pentaprion*	x	
199	*Anolis sagrei*	x	
200	*Anolis semilineatus*	x	
201	*Anolis shrevei*	x	
202	*Anolis tropidogaster*	x	
203	*Anomalopus brevicollis*	x	
204	*Anomalopus leuckartii*	x	
205	*Aphaenogaster fulva*	x	
206	*Argopecten purpuratus*	x	
207	*Arizona elegans*	x	
208	*Aulacomya atra*	x	
209	*Basiliscus vittatus*	x	
210	*Bassiana platynota*	x	
211	*Bullia rhodostoma*	x	
212	*Calyptotis temporalis*	x	
213	*Camponotus americanus*	x	
214	*Camponotus castaneus*	x	
215	*Camponotus chromaiodes*	x	
216	*Camponotus novaeboracensis*	x	
217	*Camponotus pennsylvanicus*	x	
218	*Carlia longipes*	x	
219	*Carlia munda*	x	
220	*Carlia pectoralis*	x	
221	*Carlia rhomboidalis*	x	
222	*Carlia schmeltzi*	x	
223	*Carlia vivax*	x	
224	*Charina bottae*	x	

225	*Chionactis occipitalis*	x
226	*Chondrodactylus angulifer*	x
227	*Chondrodactylus bibronii*	x
228	*Clupea harengus*	x
229	*Cnemidophorus sexlineatus*	x
230	*Coluber constrictor*	x
231	*Cophixalus bombiens*	x
232	*Cophixalus mcdonaldi*	x
233	*Cophixalus neglectus*	x
234	*Crangon crangon*	x
235	*Crinia signifera*	x
236	*Crotalus atrox*	x
237	*Crotalus cerastes*	x
238	*Crotalus scutulatus*	x
239	*Crotalus viridis*	x
240	*Crotaphytus collaris*	x
241	*Cryptoblepharus litoralis*	x
242	*Cryptoblepharus plagiocephalus*	x
243	*Cryptoblepharus virgatus*	x
244	*Ctenophorus cristatus*	x
245	*Ctenophorus fordi*	x
246	*Ctenophorus maculosus*	x
247	*Ctenophorus nuchalis*	x
248	*Ctenophorus pictus*	x
249	*Ctenophorus reticulatus*	x
250	*Ctenotus essingtoni*	x
251	*Ctenotus pantherinus*	x
252	*Ctenotus spaldingi*	x
253	*Cyclodomorphus branchialis*	x
254	*Cyclodomorphus melanops*	x
255	*Cyclorana alboguttatus*	x
256	*Cyclorana brevipes*	x
257	*Cyrtodactylus philippinicus*	x
258	*Desmognathus ochrophaeus*	x
259	*Desmognathus quadramaculatus*	x
260	*Diplodus bellottii*	x
261	*Diplodus sargus*	x
262	*Diplodus vulgaris*	x
263	*Distaplia occidentalis*	x
264	*Donax serra*	x
265	*Echinometra lucunter*	x
266	*Elgaria coerulea*	x
267	*Ensis macha*	x
268	*Eucidaris tribuloides*	x
269	*Eulamprus murrayi*	x
270	*Eulamprus tenuis*	x

271	*Eurycea longicauda*	x
272	*Eurycea lucifuga*	x
273	*Eurycea multiplicata*	x
274	*Eurycea nana*	x
275	*Floridichthys carpio*	x
276	*Formica neogagates*	x
277	*Formica obscuripes*	x
278	*Gemma gemma*	x
279	*Glaphyromorphus punctulatus*	x
280	*Gobius cobitis*	x
281	*Gobius niger*	x
282	*Gopherus agassizii*	x
283	*Heloderma suspectum*	x
284	*Hemiergis quadrilineatum*	x
285	*Holbrookia maculata*	x
286	*Hyla aurea*	x
287	*Hyla caerulea*	x
288	*Hyla chloris*	x
289	*Hyla citropa*	x
290	*Hyla lesueuri*	x
291	*Hyla phyllochroa*	x
292	*Hylarana erythraea*	x
293	*Kaloula kalingensis*	x
294	*Lacerta schreiberi*	x
295	*Lampropeltis zonata*	x
296	*Laternula elliptica*	x
297	*Lerista bougainvilli*	x
298	*Lerista frosti*	x
299	*Lerista neander*	x
300	*Lerista orientalis*	x
301	*Lerista terdigitata*	x
302	*Limatula hodgsoni*	x
303	*Limnodynastes dorsalis*	x
304	*Limnodynastes fletcheri*	x
305	*Limnodynastes tasmaniensis*	x
306	*Limnonectes woodworthi*	x
307	*Liocarcinus marmoreus*	x
308	*Liothyrella neozelanica*	x
309	*Liothyrella uva*	x
310	*Lipinia pulchella*	x
311	*Lipophrys trigloides*	x
312	*Litoria gracilenta*	x
313	*Masticophis lateralis*	x
314	*Metacarcinus magister*	x
315	*Morethia butleri*	x
316	*Morethia lineoocellata*	x

317	*Morethia obscura*	x
318	*Morethia taeniopleura*	x
319	*Mulinia lateralis*	x
320	*Mya arenaria*	x
321	*Neobatrachus pictus*	x
322	*Niveoscincus coventryi*	x
323	*Notophthalmus viridescens*	x
324	*Occidozyga laevis*	x
325	*Pachygrapsus marmoratus*	x
326	*Parablennius marmoreus*	x
327	*Parvoscincus decipiens*	x
328	*Patiriella brevispina*	x
329	*Pedioplanis lineoocellata*	x
330	*Pegusa lascaris*	x
331	*Petrosaurus mearnsi*	x
332	*Philoria frosti*	x
333	*Philoria loveridgei*	x
334	*Phrurolithus festivus*	x
335	*Phrynosoma coronatum*	x
336	*Phrynosoma platyrhinos*	x
337	*Phyllorhynchus decurtatus*	x
338	*Pinoyscincus abdictus*	x
339	*Pinoyscincus jagori*	x
340	*Pituophis catenifer*	x
341	*Platymantis banahao*	x
342	*Platymantis corrugatus*	x
343	*Platymantis dorsalis*	x
344	*Platymantis luzonensis*	x
345	*Plestiodon obsoletus*	x
346	*Plestiodon skiltonianus*	x
347	*Plethodon glutinosus*	x
348	*Podarcis bocagei*	x
349	*Podarcis tiliguerta*	x
350	*Pogonomyrmex barbatus*	x
351	*Pogonomyrmex rugosus*	x
352	*Proceratium silaceum*	x
353	*Pseudacris triseriata*	x
354	*Pseudechinus huttoni*	x
355	*Pseudophryne corroboree*	x
356	*Pseudophryne dendyi*	x
357	*Pseudotriton ruber*	x
358	*Ptenopus garrulus*	x
359	*Rhinocheilus lecontei*	x
360	*Saiphos equalis*	x
361	*Saproscincus basiliscus*	x
362	*Saproscincus tetradactyla*	x

363	*Sceloporus grammicus*	x	
364	*Sceloporus magister*	x	
365	*Sceloporus woodi*	x	
366	*Scelotes gronovii*	x	
367	*Sterechinus neumayeri*	x	
368	*Storeria occipitomaculata*	x	
369	*Tellina tenuis*	x	
370	*Tetramorium caespitum*	x	
371	*Tiliqua scincoides*	x	
372	*Trachylepis capensis*	x	
373	*Trachymyrmex septentrionalis*	x	
374	*Trematomus bernacchii*	x	
375	*Trematomus hansoni*	x	
376	*Tropidophorus queenslandiae*	x	
377	*Typhlacontias brevipes*	x	
378	*Uma inornata*	x	
379	*Urosaurus ornatus*	x	
380	*Varanus gouldii*	x	
381	*Xantusia henshawi*	x	
382	*Zoarces viviparus*	x	
383	*Acris crepitans*		x
384	*Boreogadus saida*		x
385	*Calathus melanocephalus*		x
386	*Craugastor fitzingeri*		x
387	*Engystomops pustulosus*		x
388	*Gambelia wislizenii*		x
389	*Hoplodactylus maculatus*		x
390	*Hyalinobatrachium fleischmanni*		x
391	*Leptodactylus pentadactylus*		x
392	*Liolaemus alticolor*		x
393	*Liolaemus altissimus*		x
394	*Liolaemus bisignatus*		x
395	*Liolaemus chiliensis*		x
396	*Liolaemus constanzae*		x
397	*Liolaemus curis*		x
398	*Liolaemus cyanogaster*		x
399	*Liolaemus eleodori*		x
400	*Liolaemus fabiani*		x
401	*Liolaemus fitzgeraldi*		x
402	*Liolaemus fuscus*		x
403	*Liolaemus jamesi*		x
404	*Liolaemus lemniscatus*		x
405	*Liolaemus leopardinus*		x
406	*Liolaemus lorenzmuelleri*		x
407	*Liolaemus monticola*		x
408	*Liolaemus nigromaculatus*		x

409	*Liolaemus nigroviridis*	x
410	*Liolaemus nitidus*	x
411	*Liolaemus ornatus*	x
412	*Liolaemus pictus*	x
413	*Liolaemus platei*	x
414	*Liolaemus pseudolemniscatus*	x
415	*Liolaemus schroederi*	x
416	*Liolaemus tenuis*	x
417	*Liolaemus vallecurensis*	x
418	*Liolaemus walkeri*	x
419	*Oligosoma maccanni*	x
420	*Pseudechis porphyriacus*	x
421	*Pseudocordylus melanotus*	x
422	*Scinax staufferi*	x
423	*Teratoscincus przewalskii*	x
424	*Thamnophis butleri*	x
425	*Thamnophis melanogaster*	x
426	*Thamnophis radix*	x
427	*Cnemidocarpa bicornuta*	
428	*Cookia sulcata*	
429	*Divaricella irpex*	
430	*Ischnochiton australis*	
431	*Petrolisthes gracilis*	
432	*Petrolisthes hirtipes*	
433	*Protothaca thaca*	
434	*Semele solida*	
435	*Spisula subtruncata*	
436	*Xantho incisus*	

Table S7. Number of species in study by class or clade.

Class or clade	*Number of species*
Reptilia	278
Amphibia	60
Actinopterygii	26
Bivalvia	21
Insecta	15
Arachnida	11
Malacostraca	8
Gastropoda	6
Echinoidea	4
Ascidiacea	2
Articulata	1
Asteroidae	1
Neogastropoda	1
Polyplaclophora	1
Rhynchonellata	1
