# Supplementary material for: Temperate species underfill their tropical thermal potentials on land
Source: Nat Ecol Evol. 2023 Nov 6;7(12):1993–2003. doi: 10.1038/s41559-023-02239-x (PMC10697837; doi:10.1038/s41559-023-02239-x)
Supplement: Supplementary file 2 — Reporting Summary [file 41559_2023_2239_MOESM2_ESM.pdf]

nature portfolio

Corresponding author(s): Nikki A. Moore

Last updated by author(s): Sep 20, 2023

Reporting Summary

Nature Portfolio wishes to improve the reproducibility of the work that we publish. This form provides structure for consistency and transparency in reporting. For further information on Nature Portfolio policies, see our Editorial Policies and the Editorial Policy Checklist.

Statistics

For all statistical analyses, confirm that the following items are present in the figure legend, table legend, main text, or Methods section.

n/a	Confirmed	
☐	☒	The exact sample size (*n*) for each experimental group/condition, given as a discrete number and unit of measurement
☒	☐	A statement on whether measurements were taken from distinct samples or whether the same sample was measured repeatedly
☐	☒	The statistical test(s) used AND whether they are one- or two-sided<br>*Only common tests should be described solely by name; describe more complex techniques in the Methods section.*
☐	☒	A description of all covariates tested
☐	☒	A description of any assumptions or corrections, such as tests of normality and adjustment for multiple comparisons
☐	☒	A full description of the statistical parameters including central tendency (e.g. means) or other basic estimates (e.g. regression coefficient) AND variation (e.g. standard deviation) or associated estimates of uncertainty (e.g. confidence intervals)
☐	☒	For null hypothesis testing, the test statistic (e.g. F, t, r) with confidence intervals, effect sizes, degrees of freedom and P value noted<br>*Give P values as exact values whenever suitable.*
☒	☐	For Bayesian analysis, information on the choice of priors and Markov chain Monte Carlo settings
☐	☒	For hierarchical and complex designs, identification of the appropriate level for tests and full reporting of outcomes
☒	☐	Estimates of effect sizes (e.g. Cohen's d, Pearson's r), indicating how they were calculated

Our web collection on statistics for biologists contains articles on many of the points above.

Software and code

Policy information about availability of computer code

Data collection: NA

Data analysis: *Provide a description of all commercial, open source and custom code used to analyse the data in this study, specifying the version used OR state that no software was used.*

For manuscripts utilizing custom algorithms or software that are central to the research but not yet described in published literature, software must be made available to editors and reviewers. We strongly encourage code deposition in a community repository (e.g. GitHub). See the Nature Portfolio guidelines for submitting code & software for further information.

Data

Policy information about availability of data

All manuscripts must include a data availability statement. This statement should provide the following information, where applicable:

- Accession codes, unique identifiers, or web links for publicly available datasets
- A description of any restrictions on data availability
- For clinical datasets or third party data, please ensure that the statement adheres to our policy

Provide your data availability statement here.

Human research participants

Policy information about studies involving human research participants and Sex and Gender in Research.

Reporting on sex and gender	N/A
Population characteristics	N/A
Recruitment	N/A
Ethics oversight	N/A

Note that full information on the approval of the study protocol must also be provided in the manuscript.

Field-specific reporting

Please select the one below that is the best fit for your research. If you are not sure, read the appropriate sections before making your selection.

☐ Life sciences ☐ Behavioural & social sciences ☒ Ecological, evolutionary & environmental sciences

For a reference copy of the document with all sections, see nature.com/documents/nr-reporting-summary-flat.pdf

Ecological, evolutionary & environmental sciences study design

All studies must disclose on these points even when the disclosure is negative.

Study description	A previously-published dataset on thermal tolerance limits of ectotherms globally was combined with data from individual previously-published studies (species traits), and queried from public sources (georeferenced species occurrence data, geographic range polygons, climate data) in order to produce derived data products (potential and realized thermal niche and thermal range). Models were fit to test a-priori hypotheses about how these values vary according to latitude, realm, and species traits.
Research sample	The sample was global marine and terrestrial ectotherms, within a previously published dataset of thermal tolerance limits from an exhaustive review (GlobTherm, Bennet et al. 2018).
Sampling strategy	We included all marine and terrestrial ectotherms within the previously published dataset of thermal tolerance limits (GlobTherm), which itself represents an exhaustive global collation of thermal tolerance limit assay data according to prescribed inclusion criteria.
Data collection	Species range occurrence data was collected by authors J.M.S. and A.L.H., who queried the Global Biodiversity Information Criterion for occurrence records and independent descriptions of species ranges. Species traits data were collected by authors N.A.M., J.M.S., I.M-C., A.L.H., M.A.O.T., F.V., P.C., S.C-T., A.C.A., B.M., L.R., S.G., and J.M.B who each completed literature searches and queried databases for body size, generation time, dispersal ability, migration type and seasonal dormancy capacity, for a subset of the species in our database. These were harmonized and quality controlled (checked for correct units and entry types) by S.G. and N.A.M. Acclimation response data was collected from previous-published studies by J.R.B.. Species' range polygons, thermal preference data, and geo-referenced climate data were queried from public databases and compiled by N.A.M.
Timing and spatial scale	Thermal tolerance limit data were included up to Oct. 2016 as described elsewhere (Bennet et al. 2018, https://doi.org/10.1038/sdata.2018.22). Species traits were collected up to Sept. of 2020. Georeferenced occurrence data were extracted from the Global Biodiversity Information Criterion on Dec. of 2016, including all time points in database. Range polygons were downloaded from IUCN (IUCN 2020) on October 1, 2020 and from the GARD Database (Roll et al. 2017). Study was global in scale.
Data exclusions	We filtered occurrence records from the Global Biodiversity Information Criterion to only include points that were within described species ranges according to an independent source to reduce the probability of spurious range estimates. All decisions about occurrence point exclusion and independent geographic range descriptions and sources will be available in tabular and script form within our published data and code. We excluded species if occurrence records had fewer than 30 point locations, or had did not represent the entire species range according to independent range descriptions, as described in our methods, and all decisions about species exclusion based on poor range descriptions will be available in tabular and script form within our published data and code.
Reproducibility	All code is available to reproduce calculations, visualizations, and analyses reported. When possible, code used for extracting records from databases is available. However we did not attempt to independently repeat manual data collation from the literature.
Randomization	Organisms were not allocated into groups for data extraction nor data collation. We did include random effects in our models to account for taxonomic non-independence among the species in the dataset, and accounted for methodological differences among studies with model covariates.
Blinding	Datasets were collated based on a priori inclusion criteria, and visualization of data aggregates were not made until datasets were complete.

Did the study involve field work? ☐ Yes ☒ No

Reporting for specific materials, systems and methods

We require information from authors about some types of materials, experimental systems and methods used in many studies. Here, indicate whether each material, system or method listed is relevant to your study. If you are not sure if a list item applies to your research, read the appropriate section before selecting a response.

Materials & experimental systems

n/a	Involved in the study
☒	☐ Antibodies
☒	☐ Eukaryotic cell lines
☒	☐ Palaeontology and archaeology
☒	☐ Animals and other organisms
☒	☐ Clinical data
☒	☐ Dual use research of concern

Methods

n/a	Involved in the study
☒	☐ ChIP-seq
☒	☐ Flow cytometry
☒	☐ MRI-based neuroimaging
